# The physician factor and anatomical site in 8846 consecutive mediastinal lymph node aspirations in a cross-sectional study

**DOI:** 10.1038/s41598-022-26962-w

**Published:** 2023-01-31

**Authors:** Michael Bonert, Uzma Zafar, Soha Ramadan, Christian Finley, Jean-Claude Cutz, Gary Foster, Kjetil Ask, Asghar Naqvi

**Affiliations:** 1 Division of Anatomical Pathology, Department of Pathology and Molecular Medicine, McMaster University, Hamilton, ON Canada; 2 Department of Pathology, St. Joseph’s Healthcare Hamilton, Room L222-3, 50 Charlton Avenue East, Hamilton, ON L8N 4A6 Canada; 3 Department of Pathology, Rutgers Health/St. Barnabas Medical Center, Livingston, NJ USA; 4 Division of Thoracic Surgery, Department of Surgery, McMaster University, Hamilton, ON L8S 4L8 Canada; 5 St. Joseph’s Healthcare Hamilton, Hamilton, ON L8N 4A6 Canada; 6 Department of Health Research Methods, Evidence and Impact, McMaster University, Hamilton, ON Canada; 7 Department of Medicine, McMaster University, Hamilton, ON L8S 4L8 Canada

**Keywords:** Respiratory tract diseases, Cancer, Diseases, Health care, Medical research, Oncology

## Abstract

Mediastinal lymph node fine needle aspiration (MLN-FNA) is a common procedure; however, the physician factor in pathological category, and anatomical site are not routinely assessed. Cytology reports for endobronchial ultrasound (EBUS)/endoscopic ultrasound (EUS) MLN-FNA specimens (8846) were retrieved for July 2012–Dec 2019, classified by hierarchical free text string match algorithm into 51 diagnostic categories, four mutually exclusive diagnostic groups (benign |suspicious |malignant |insufficient), and 24 anatomical sites. Pathologist and submitting physician/surgeon bias were assessed using logistic regression and funnel plots|control charts centered on the group median (diagnostic/capture) rate. Eleven pathologists and seven submitting physician/surgeon were involved in more than 250 specimens each. Overall, the MLN-FNAs were benign|suspicious|malignant|insufficient in 46%|4%|25%|24% of specimens. Percent malignant (number of samples) varied by station; 7| 4R| 4L| 2R| 10R| 11R| 11L were respectively 21%(3,101), 27%(2,453), 19%(1,289), 41%(435), 27%(497), 24%(357), 26%(229). The number of outlier (*P* < 0.05/*P* < 0.001) pathologists of 11 from the group median rate for benign|suspicious|malignant|insufficient was 0/0| 3/1| 0/0| 3/0 respectively. The outlier (*P* < 0.05/*P* < 0.001) submitting physicians/surgeons of 7 for benign|suspicious|malignant|insufficient was 3/2| 2/2| 3/2| 3/2 respectively. The physician and anatomical site are significant predictors of MLN-FNA pathology.

## Introduction

The concept of real time endobronchial ultrasound guided Transbronchial Needle Aspiration (EBUS-TBNA) was introduced in 1997, and the potential use of EBUS-TBNA in mediastinal staging was published for the first time in 2002^1^. It is an amalgamation of traditional bronchoscopy, ultrasonography, and needle aspiration cytology.

It is important to realize that the initial diagnostic procedures for lung cancer should simultaneously provide tissue for histopathological diagnosis, molecular testing, and staging^2^. EBUS is more sensitive and specific than radiological techniques, as tissue samples can be taken for pathological assessment. The gold standard method for invasive mediastinal lymph nodes (LNs) staging in lung cancer was mediastinoscopy. However, the introduction of the convex probe, in the early 2000s, has transformed the technique of mediastinal LN staging in lung cancer, as it allows real-time TBNA of the visualized tissue^3^.

The accessibility of endobronchial ultrasound (EBUS) is similar to mediastinoscopy for 2R, 2L, 4R, 4L and 7 LN^4^. The accessibility of EBUS is superior to mediastinoscopy for posterior and deeply located station 7 LNs^5^. EBUS-TBNA can reach N1 LNs (including stations 10, 11 and 12) which are not accessible by mediastinoscopy^4,5^.

Mediastinoscopy is an invasive procedure performed in an operating room under general anesthesia. The complication rate is 2.5%^4^ and mortality rate is 0.08%^6^. Whereas EBUS/ EUS is a relatively safe procedure performed in endoscopy suite under conscious sedation. The complication rate is 1.23%^5^ and mortality is 0.01%^7^.

The diagnostic accuracy of EBUS is increased by adding transesophageal endoscopic ultrasound- fine needle aspiration (EUS- FNA) as a complementary technique. EUS is preferred over EBUS at station 7 and 4L^8^.

The American College of Chest Physicians (ACCP) and the European Society of Thoracic Surgeons (ESTS) have made following recommendations^4^ regarding the tests of first choice in invasive mediastinal LN staging:

1. Endoscopic mediastinal staging with EBUS-TBNA or esophageal ultrasound fine needle aspiration (EUS-FNA)
2. In case of negative results, and if the index of suspicion for metastatic disease is high, mediastinoscopy should be performed.

The supremacy of endobronchial needle aspiration (EBNA) over other techniques is now established^4^. Although the technical aspects of the EBUS have been studied in great detail, statistical evaluation of the expertise of submitting physicians and diagnostic accuracy of the reporting pathologist is lacking. The physician is a well-known predictor of medicinal and surgical outcomes; however, this is not routinely assessed in a large number of contexts.

### Objective

The goal of this work is to assess the physician factor and anatomical site in the context of consecutive mediastinal lymph node fine needle aspirations (MLN-FNA) at a large lung referral center. Based on prior work, we hypothesize the physician factor and anatomical site are significant predictors of the diagnostic classification.

## Methods

The study was conducted at St. Joseph’s Healthcare Hamilton after research ethics board (Hamilton Integrated Research Ethics Board (HiREB) approval to analyze the data (HiREB# 3811). The study was done in accordance with national ethics guidelines and relevant regulations. This study had no research subjects; thus, the requirement for informed consent from subjects is not applicable.

All cytopathology results for endobronchial ultrasound (EBUS)/endoscopic ultrasound (EUS) MLN-FNA specimens were retrieved from the Laboratory Information system (MEDITECH) for a period of 7.5 years (July 2012–December 2019) and analyzed with a set of programs that have previously been used to analyze a number of specimen types^9^. A summary of the methods is provided in flow chart form in Appendix M.

Patient identifiers were removed and the medical record number was replaced with a unique identifier. The physicians were anonymized. Cases were classified and the physician rates tabulated. Statistical analyses were done using R (statistical software)^10^ (https://cran.r-project.org), and SAS (https://www.sas.com/en_us/home.html). Funnel plots and control charts were generated for visual representations of the data.

Criteria for inclusion into the study were that:

1. The case was an in-house cytopathology case, and.
2. The “source of specimen” section of the pathology report contained all the following:
  - “lymph node”.
  - “EBUS” or “EUS”.

Fifty-one diagnostic categories were created and linked with 387 search terms/phrases listed in Supplemental A. Similarly, 24 anatomical location categories were identified and linked to 50 search terms/phrases shown in Supplemental B. The anatomical locations are the ones used by the *International Association of the Study of Lung Cancer/American Joint Committee on Cancer*. The categorization dictionaries were constructed from a list of diagnoses and refined via iteration: (1) results were generated, (2) accuracy assessed, (3) dictionary refined, (4) process 1–3 repeated until accuracy deemed acceptable.

Diagnostic categorical hierarchies, which make use of logic and reporting structures, were defined. In this way, “non-small cell carcinoma” and (the sub-string) “small cell carcinoma” can be reasonably well separated. Non-specific categories, such as “non-small cell carcinoma, favor adenocarcinoma” were classified as “adenocarcinoma”, as this is how it is generally interpreted.

Diagnostic classification was based on the content of the ‘diagnosis’ section, the ‘consultation’ section and all report addenda if present.

Diagnostic classifications were grouped into four categories: insufficient, benign, suspicious, and malignant. These groupings were considered mutually exclusive, and ordered within the following hierarchy: malignant, suspicious, insufficient, and benign. How the groups relate to the diagnostic categories is shown in Supplemental C.

The analysis builds on prior work, which assumes that: (1) individual patients are assigned randomly to providers and that pathologists are randomly assigned individual cases, and (2) disease characteristics are approximately stable within the comparison intervals.

Pathologist and submitting physician/surgeon bias were assessed using logistic regression (LR) and funnel plots/control charts. Funnel plots and control charts were centered on the group median (diagnostic/capture) rate (GMR). To avoid the possibility of over-fitting when modeling with logistic regression, physicians/surgeons submitting/interpreting less than 250 specimens were excluded as were anatomical locations with less than 20 records.

## Results

The study period contained 8846 lymph node specimens of which 98.4% could be placed in one of the four mutually exclusive diagnostic categories.

Five hundred cases were randomly selected and audited by pathologists to assess the accuracy of the hierarchical free text string matching algorithm (HFTSMA). Of these cases 99% were categorized and 99% of categorized cases were correctly categorized. We were unable to determine a diagnostic classification for 0.7% of cases in our study. Based on prior experience with the HFTSMA, the un-categorized cases are likely to be the result of (1) ambiguously worded reports (difficult for a pathologist to classify), (2) descriptive diagnoses, (3) reports that could not be classified due to report formatting, and (4) a small group of cases where the HFTSMA fails due to an unknown reason. A sub-analysis of the ‘metastasis’ category (57 cases) had an error rate of about 5%.

One or more addenda or a formal consult was attached to the report in 869 cases; these changed the diagnosis of 166 cases. The most frequent change was non-small cell carcinoma to adenocarcinoma (77 cases), followed by non-small cell carcinoma to squamous cell carcinoma (21 cases), malignant not further specified to small cell carcinoma (13 cases), and malignant not further specified to adenocarcinoma (13 cases). A mix of different changes were seen in 44 cases; the largest subgroup was a change to lymphoma (12 cases). Examples of cases with their classification are given in Appendix B.

Eleven pathologists each interpreted more than 250 specimens (range 331–1,024) and together they read 7931 specimens. Seven physicians/surgeons submitted more than 250 specimens each (range 467–2196) and together obtained 8760 specimens.

Seen from the perspective of pathology, 2R is the most malignant node; when it is sampled, it is the most likely to be malignant. The N1 lymph nodes and station 4L specimens have higher insufficiency rates; this was an expected finding. N1 lymph nodes are in the distal bronchial tree and more difficult to access. Station 4L likewise is technically more difficult to reach (Tables 1 and 2).

**Table 1:** Lymph node cohort by diagnosis and mutually exclusive grouping.

Diagnosis	Number	Fraction
**Diagnoses and diagnostic groups – overview diagnoses (selected)**		
Adenocarcinoma	749	0.085
Squamous carcinoma	282	0.032
Small cell carcinoma	344	0.039
Non-small cell carcinoma	469	0.053
Carcinoma not otherwise specified	210	0.024
Metastasis	291	0.033
Granulomatous inflammation	696	0.079

Exclusive group	Number	Fraction
**Mutually exclusive groupings**		
Benign	4059	0.459
Suspicious	375	0.042
Malignant	2194	0.248
Insufficient	2152	0.243
Unclassified	66	0.007
*All cases*	*8846*	*1.000*

Note: All cases in itlaics was put in italics to emphasize that it is a summation of all the above mentioned 5 mutually exlusive categories.

**Table 2:** Lymph node cohort by mutually exclusive grouping and anatomical location (selected).

Group\Station	Station 7	Station 4R	Station 4L	Station 2R	Station 10R	Station 11R	Station 11L	All cases
**Lymph Node Fine Needle Aspiration by Station (Selected) and Diagnostic Category**								
Benign	0.527	0.493	0.419	0.3	0.374	0.345	0.362	0.459
Suspicious	0.037	0.039	0.043	0.053	0.05	0.048	0.07	0.042
Malignant	0.208	0.268	0.187	0.413	0.274	0.238	0.262	0.248
Insufficient	0.222	0.19	0.346	0.231	0.296	0.356	0.297	0.243
Unclassified	0.006	0.01	0.005	0.003	0.006	0.013	0.009	0.008
Insuff. + Susp.	0.259	0.229	0.389	0.284	0.346	0.404	0.367	0.285
Number	3101	2453	1289	433	497	357	229	8846
Fraction	0.351	0.277	0.146	0.049	0.056	0.040	0.026	1.000

The main rate differences among the pathologists (in the sub-classification of cancers—see Table 3) is likely driven by interpretative factors.

**Table 3:** Logistic regression results in cohort for mutually exclusive grouping and diagnosis (selected) with the predictors: SPS, Pathologist, and Station (Location).

Category\factor	Submitting physician/surgeon (SPS)	Pathologist	Station (Location)
**Logistic regression results for diagnostic category/selected diagnoses**			
Degrees of freedom	6	10	12
Benign	< 0.0001	0.5652	< 0.0001
Suspicious	0.0428	< 0.0001	0.2796
Malignant	< 0.0001	0.1072	< 0.0001
Insufficient	< 0.0001	0.0826	< 0.0001
Adenocarcinoma	0.0570	0.0012	< 0.0001
Squamous cell carcinoma	0.1776	0.2080	0.0542
Non-small cell carcinoma	0.0007	< 0.0001	0.0016
Small cell carcinoma	< 0.0001	0.0059	0.0009

### Funnel plots and control charts

Funnel plots and control charts were generated for the mutually exclusive categories (benign, suspicious, malignant, insufficient) and included all pathologists who interpreted more than 250 specimens. How the control charts were generated is described in Appendix A.

**Figure 1:**
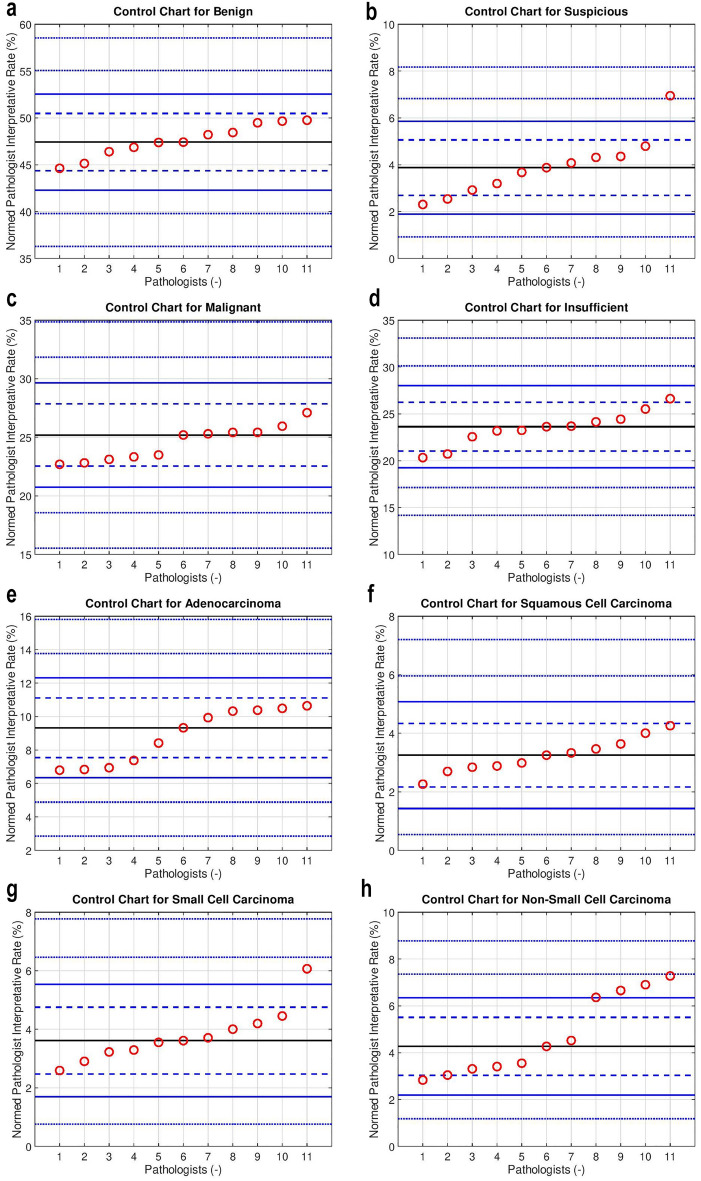
Pathologist control charts for the mutually exclusive diagnostic categories ((**a**) benign, (**b**) suspicious, (**c**) malignant, (**d**) insufficient), and the main subtypes of cancer ((**e**) adenocarcinoma, (**f**) squamous cell carcinoma, (**g**) small cell carcinoma, (**h**) non-small cell carcinoma. The red circles represent individual pathologists. Pathologists are ordered by the normalized diagnostic rate. The black line in the centre of the control chart is the group median diagnostic rate (GMDR). The dashed blue (control) lines represent the boundaries of the 95% confidence interval (CI) and correspond to *p* = 0.05. The solid blue (control) lines represent the boundaries of the 99.9% CI and correspond to p = 0.001. The dashed blue (control) lines beyond the solid blue line represent the *p* = 1e-6 and *p* = 1e-12 respectively. Details of the normalization are in Appendix A. It should be noted that: each control chart is sorted by the rate; pathologist #1 on one chart is generally not pathologist #1 on any other chart.

The control charts (see Fig. 1a–d) show that the pathologist diagnostic rates differ significantly (*P* < 0.001) for the suspicious category. In contrast, the pathologists’ rates vary modestly for insufficient and benign (no *P* < 0.001 outliers), and the malignancy rates differ minimally (there are no *P* < 0.05 outliers).

The major cancer subtypes (adenocarcinoma, squamous cell carcinoma, small cell carcinoma) are presented as control charts (see Fig. 1e–g). Squamous cell carcinoma has no statistical outliers (see Fig. 1f). Non-small cell carcinoma, adenocarcinoma and small cell carcinoma show some diagnostic rate variation (see Fig. 1h, e and g).

**Figure 2:**
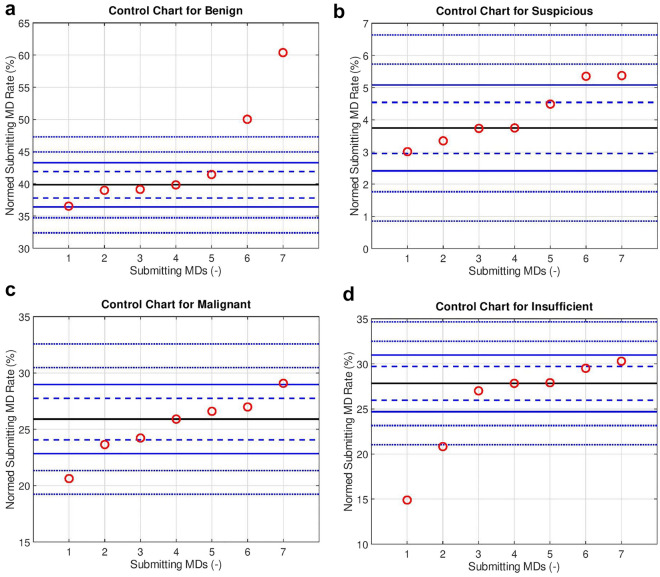
Submitting MD control charts for the mutually exclusive diagnostic categories ((**a**) benign, (**b**) suspicious, (**c**) malignant, and (**d**) insufficient). The red circles represent individual submitting MDs. Submitting MDs are ordered by the normalized rate. The black line in the centre of the control chart is the group median rate (GMR). The dashed blue (control) lines represent the boundaries of the 95% confidence interval (CI) and correspond to p = 0.05. The solid blue (control) lines represent the boundaries of the 99.9% CI and correspond to p = 0.001. The dashed blue (control) lines beyond the solid blue line represent the *p* = 1e-6 and *p* = 1e-12 respectively. Details of the normalization are in Appendix A. It should be noted that: each control chart is sorted by the rate; submitting MD #1 on one chart is generally not submitting MD #1 on any other chart.

The submitting MDs were examined with control charts for mutually exclusive diagnostic categories; benign, suspicious, malignant and insufficient (see Fig. 2a–d). These show that there are statistical outliers for all mutually exclusive categories.

### Logistic regression (LR)

In order to avoid the possibility of overfitting, rare locations (present in < 20 specimens) and low volume pathologists and submitting physicians/surgeons (those submitting/interpreting < 250 specimens) were excluded from the analyses. This resulted in a trimmed data set with 7,777 specimens, 7 SPS (submitting physicians/surgeons), 11 pathologists, and 13 anatomical locations (nodal stations).

Results from LR modeling (see Table 3) largely mirror those seen in the funnel plots/control charts but are not identical. Submitting physician/surgeon (SPS) is a significant predictor of benign, malignant and insufficient (all *P* < 0.0001) categories. Suspicious is a pathologist driven category (*P* < 0.0001) and mildly influenced by SPS (*P* = 0.0433). Insufficient is a SPS driven category (*P* < 0.0001) without significant differences among pathologists (*P* = 0.0831). Anatomical location (nodal station) is a strong predictor (*P* < 0.0001) of benign, malignant and insufficient; it is not significantly predictive of suspicious (*P* = 0.2796).

Modeling results for specific diagnostic categories (adenocarcinoma, squamous cell carcinoma and small cell carcinoma (SCC) are mixed (see Table 3). The individual SPS is a significant predictor of small cell carcinoma (*P* < 0.0001). The individual pathologist is a strong predictor of non-small cell carcinoma (*P* < 0.0001), and a moderate predictor of adenocarcinoma (*P* = 0.0012) and small cell carcinoma (*P* = 0.0059). The latter prompted a targeted review that suggested the findings were driven by case assignment/an unlikely random occurrence rather than interpretative.

The logistic regression results were used to generate receiver operator characteristic curves. These are in the supplemental materials and show visually (1) that most of the variation is intrinsic to the specimen, and (2) how the independent variables (SPS, pathologist, location) relate to one another.

## Discussion

The management of lung cancer starts with the categorization of histological type and stage of the tumor. Although there are several techniques, the most recent and minimal invasive technique is EBUS, alone or in combination with EUS. We have an enormous amount of medical data at our disposal yet extracting the right information is vital for making meaningful inferences about it. Most of the information communicated by the physicians is available in the free text form and not coded so manually retrieving the relevant data for research purposes is a tedious, expensive and time-consuming task. Shah et al.^11^ in 2012 wrote a computer program- “Free Text Matching Algorithm” and used it to extract information from the UK General Physician Database to analyze the cause of death.

In our study, we set up a similar program to extract information from the free text based on the key “words” and “phrases” we generated. An exhaustive table (Supplemental A) which includes all the possible medical terms and their combinations used by pathologists in reports was created for the precise categorization of cases. Supplemental B contains all the anatomical location codes and search strings. We audited 500 specimens using HFTSMA and it could assign an anatomical site and diagnosis in 99% cases and the coding was accurate 99% of the time. It is possible that other text processing approaches, such as machine learning, may yield a superior categorization; however, in the context of the (healthcare provider) effect size measured, refinement would not substantially change the conclusions. The limitations of HFTSMA could be overcome by having structured report formats and categorical diagnoses; these would reduce the inter-personal variations in pathology reporting.

Our analysis establishes that the LN location, the SPS and the pathologist are independent variables determining the outcome of EBUS/EUS for diagnosis and staging of Lung cancer.

The analysis of 8846 cases showed that the proportion of benign cases (0.459) was significantly more than that of malignant cases (0.248) or insufficient cases (0.243). (see Table 1) The suspicious cases constituted a smaller proportion (0.042). Nayak et al.^12^ reported these figures as 1% suspicious, 42% malignant and 28% insufficient cases; whereas Jeebun and Harrison^13^ reported 1.1% suspicious, 50.7% malignant, and 3.6% inadequate cases.

We went a step further to calculate these fractions for individual LN stations. We found that the distribution was not uniform for all the lymph node stations. When combined, the proportion of insufficient and suspicious cases was highest for station 11R (0.404) and lowest for 4R (0.229)**.** To the best of our knowledge, this is the first study of its kind looking at the distribution of cases into diagnostic categories at each LN station. (see Table 2).

The variation of distribution from one station to another could possibly be explained by the variation in location and histological type of the primary tumor. Another possible explanation could be the preferential involvement of different LN stations by various disease processes. This underscores the possibility of a unique benign- malignant ratio for each station.

We suspect that there may be an optimal malignancy/(benign + malignant) rate for each nodal station that would ensure a high sensitivity for malignancy without excessive resource use. A submitting physician/surgeon that has a higher malignancy rate may be under-sampling the station and missing positives. The converse may also be true; a physician/surgeon that has a high benign rate may be safely able to forego sampling a given node without missing a positive and thereby increase their efficiency.

Due to the complex anatomy of the mediastinum, some of the lymph nodes stations are accessible by EBUS and others by EUS. The most common station sampled by EBUS/EUS was station 7 (0.351 fractions of cases), and combined station 7, 4R and 4L constituted more than 77% of the samples, which has been reported as 72.6%^13^ and 72%^14^ in other studies.

The insufficient and suspicious cases combined constitute about a third of the cases (29%), thus suggesting a need for another diagnostic procedure for such cases.

Results from the LR analysis show that “suspicious” is a category significantly associated with pathologists. Out of 8,846 cases, 375 cases were diagnosed as suspicious which is only 4.2%. Skov et al.^15^ studied inter-observer variation for suspicious cases on EBUS-TBNA and concluded that the reproducibility of a diagnostic test is directly proportional to the experience of a pathologist. However, pathologists with poor reproducibility for suspicious cases on EBUS-TBNA samples showed marked improvement after some education. We also found significant variation in the “suspicious” category which we believe could be improved with follow-up, further training, and sub-specialty sign-out.

LR results show that “insufficient” is a category significantly predicted by SPS and lymph node stations. Insufficient sampling can be related to the expertise of the submitting physician. To acquire basic competency in EBUS, a trainee needs to perform at least 50 procedures under supervision and to maintain the competency the operator should perform at least 20 EBUS every year^16^. EBUS learning curves plotted by Christina et al.^14^ showed a positive correlation between operator training and positive yield. A noteworthy point is that the increase in the yield was due to fewer unsatisfactory samples.

EBUS-TBNA is a multi-step procedure hence several factors can potentially affect sample adequacy, such as skill and training of SPS, lack of uniformity in technique, needle size used, type of sedation, lymph node size and location.

An adequacy score for EBUS-TBNA samples was proposed by Alsharif et al.^17^ based on the number of lymphocytes per high power field, however it was found too stringent by other authors^12,18^. Nayak et al.^12^ proposed that “any smear with more than 5 low power fields (10 objective; X100 magnification, area not specified) of at least 100 lymphocytes each and containing less than 2 groups of bronchial cells/low power field (10X objective; X100 magnification) can be considered adequate for evaluation. Another adequacy criterion was the presence of germinal center fragment by itself irrespective of the aforementioned criteria. At our institution, we use a modified Nayak criterion of 500 lymphocytes or at least one germinal center.

ROSE (Rapid on-site evaluation) is used for increasing adequacy rate and also decreasing EBUS/EUS FNA time. It also provides onsite preliminary diagnosis especially in cases which have inconclusive macroscopic appearance^19^. ROSE is used selectively in our environment and usage was not captured on the pathology requisition; thus, it was not part of the analysis herein. It’s mainly used in our institution for adjudicating the adequacy of the specimen.

Other authors^20^ have proposed that the sensitivity and accuracy rates of ROSE slides were increased when the size of tissue core was large (≥ 2 cm), microscopic anthracotic pigment (MAP) was present, and with increased lymphocyte density (LD; ≥ 40 cells/field [40 × , mean of 10 fields]).

EBUS/EUS MLN-FNA, when compared to other techniques (such as mediastinoscopy), should consider the effect of the physician factor, as it may be a significant confounder.

In few practice environments SPS/pathologists are aware of their capture rates/call rates. This may represent a missed opportunity to understand where effort to improve quality can be directed, especially when the data is visualized within control charts (or funnel plots).

Control charts are used within manufacturing for process management and optimization, and a component of statistical process control^21^. They are infrequently used in medicine; however, they may be accessible to physicians with a small amount of effort. Control charts (also known as Shewhart charts), can (1) provide feedback to the individual physicians about their performance (in relation to their peers), and (2) facilitate comparisons between practice environments. Control charts (and funnel plots) allow one to easily identify statistical outliers and provide information on effect size and direction (higher rate/lower rate to group median call rate).

The control charts herein show how the rates of the pathologists and SPS compare in each of the 4 diagnostic categories. The individual pathologist’s diagnostic rates (DR) deviation from the median was maximum for the “suspicious” and least for the “malignant”. The variation was modest for the “benign” and the “insufficient”. To the best of our knowledge, no study has been conducted so far to compare the diagnostic rates of individual pathologists for EBUS/EUS samples.

The individual SPS diagnostic rate variance is present in all 4 categories. This is in keeping with prior work; the skill and experience of the SPS, the lymph node location and size; and the type of sedation used for the procedure are some of the factors deciding the diagnostic yield^21^.

Our non-diagnostic/unsatisfactory rate is towards the higher end of what is reported; the Non-Diagnostic rate varies from 7 to 27% in the literature^22^. However, we have noted a decreasing trend in the unsatisfactory rate over time.

EBUS simulators are used to help train MDs in tissue acquisition^23,24^. Simulation may be a tool that could be used to help understand the submitting MDs rate differences.

There are no standardized categories for specifically reporting EBUS/EUS-FNA cytology cases.

Our literature search found a significant variation in the reporting categories. Non-standardization of reports may lead to report misinterpretation to unnecessary duplication of work (in the form of consultations) and unneeded auxiliary tests.

Several different site-specific cytology specimens have recommended reporting categories notable amongst them are the Bethesda system for the Cervical cytology, Milan system for Salivary gland specimens, Paris system for Urine cytology, and Papanicolaou for Pulmonary cytopathology.

The authors feel that the EBUS-FNA reporting shares come common grounds with the Papanicolaou system for the Pulmonary specimens but has its own unique nature which justifies using a different terminology of its own.

We propose a simplified categorization scheme with four categories – see Table 4.

**Table 4:** Proposed pathological classification with four main categories and six subcategories.

Category	Subcategories
**Proposed Pathological Classification for Mediastinal Lymph Nodes**	
Benign lymph node	Reactive changes, Granulomatous inflammation, Other
Suspicious for malignancy	–
Malignant	Pulmonary, Non-Pulmonary, Other
Unsatisfactory/Insufficient	–

We feel that the “atypical category” does not add value to the above-mentioned categories.

The EBUS-FNA is mostly used for sampling of lymph nodes and infrequently for tumor/lesion sampling. Having lesser categories leads to simplified interpretation and less management challenges.

We created categorical variables from free text. It is easier to do this analysis if the reporting is standardized.

We propose that the pathological classification should be standardized (see Table 4). Categorical data (as opposed to free text) would considerably simplify the analysis herein and may avoid or reduce the ‘unclassified’ group that represented ~ 0.7% of the data set.

The process herein is largely automated and scales favorably with case volume. The potential usefulness of this approach increases with volume/the size of practice. If the volume is too low the physician rates are poorly estimated.

The size of difference that can be detected is dependent on the width of the funnel chart/control chart.

The insufficient rate uniformity among pathologists and variance among submitting physicians/surgeons (SPS) in the multivariate analysis is in keeping with SPS differences in performance. The effect size can be ascertained from the rate differences on the control chart (see Fig. 2d). A sub-analysis (data available online) suggests that the data may be able to guide SPS improvement efforts, e.g. a SPS could be directed to focus their efforts on one or more nodal stations that in relative terms has more insufficient cases.

## Limitations

The HFTSMA has a limited accuracy and may not detect small differences. Systematic reporting classifications errors may occur due to unusual report formatting or diagnostic language; this would be avoided if the pathological classifications were categorical.

The human audits identified at least one systematic misclassification error, that resulted from diagnostic wording that was used by one pathologist. The error shifted the diagnostic rates 1–2%; it was corrected. The possibility of another similar error is deemed unlikely; however, it cannot be completely excluded. We are certain that the overall conclusions would not change.

## Conclusions

The pathologist, submitting physician/surgeon and the anatomical location (of sampled lymph nodes) are significant predictors of diagnostic classification. Significant associations between predictor variables and diagnostic classification were observed that are statistically unlikely to be due to chance. These associations are suspected to be due to physician/surgeon/pathologist training or biases/aptitude. These findings suggest that there is room for improvement among submitting physicians/surgeons/pathologists. We plan to use the data herein to further continuous quality improvement and can foresee that observational data will increasingly shape medical practice.

## Supplementary Information

Supplementary Information 1.

Supplementary Information 2.

Supplementary Information 3.

Supplementary Information 4.

Supplementary Information 5.

Supplementary Information 6.

## Data Availability

The datasets generated during and/or analysed during the current study are available from the corresponding author.
