# Supplementary material for: The physician factor and anatomical site in 8846 consecutive mediastinal lymph node aspirations in a cross-sectional study
Source: Sci Rep. 2023 Jan 31;13:1784. doi: 10.1038/s41598-022-26962-w (PMC9889352; doi:10.1038/s41598-022-26962-w)
Supplement: Supplementary file 1 — Supplementary Information 1. [file 41598_2022_26962_MOESM1_ESM.docx]

**Appendix A – Creating Control Charts**

The normal approximation of the binomial distribution:


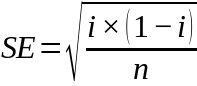
 Equation C1

Where:

SE = standard error

i = ideal (diagnostic) rate

n = number of specimens interpreted

The physician rate (e.g. pathologist diagnostic rate) is normed as follows:


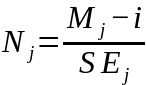
 Equation C2

Where:

N_j_ = physician rate for physician “j”

M_j_ = measured rate for the physician “j”

i = ideal (diagnostic) rate

SE_j_ = SE for physician “j”

Equation C2 can be substituted into Equation C1:


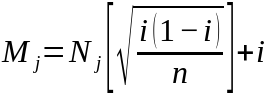
 Equation C3

To norm it was presumed that the “SE” is equivalent and that only “n” changes. This amounts to forming two equations from Equation C3 and solving for the normed M_j_. After some rearrangement one can derive an equation to convert between ‘n’ values:


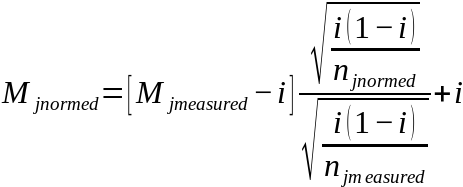
 Equation C4

Where:

M_j normed_ = normed (diagnostic) rate for physician “j”

M_j measured_ = measured (diagnostic) rate for physician “j”

n_j normed_ = normed number of specimens handled (interpreted) by physician “j”

n_j measured_ = number of specimens handled (interpreted) by physician “j”

i = ideal (diagnostic) rate
