# Supplementary material for: The physician factor and anatomical site in 8846 consecutive mediastinal lymph node aspirations in a cross-sectional study
Source: Sci Rep. 2023 Jan 31;13:1784. doi: 10.1038/s41598-022-26962-w (PMC9889352; doi:10.1038/s41598-022-26962-w)

Appendix M
The Physician Factor and Anatomical Site in Mediastinal Lymph Node Aspirations in a Cross-Sectional Study

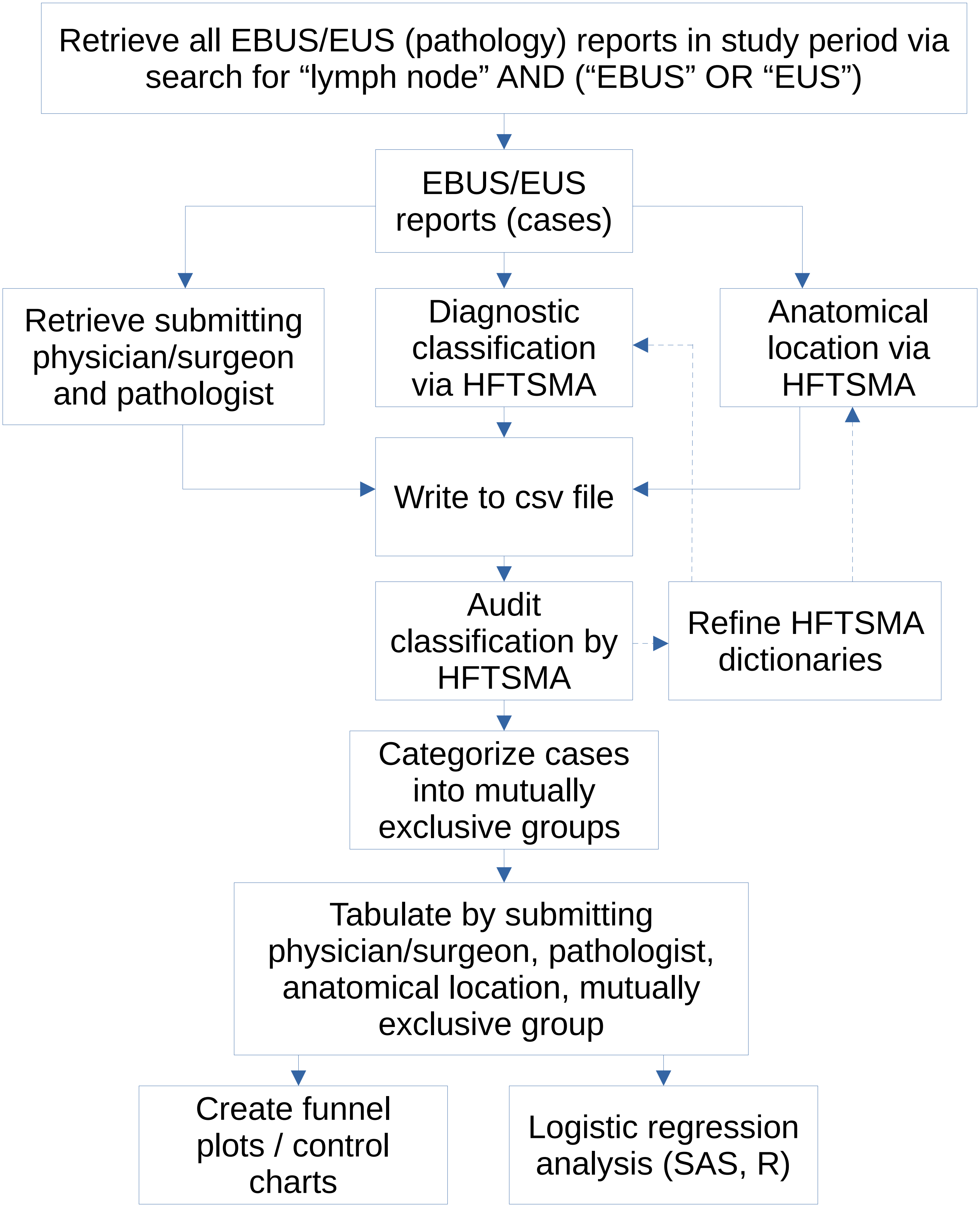

Supplement: Supplementary file 3 — Supplementary Information 3. [file 41598_2022_26962_MOESM3_ESM.pdf]
