# Supplementary material for: The physician factor and anatomical site in 8846 consecutive mediastinal lymph node aspirations in a cross-sectional study
Source: Sci Rep. 2023 Jan 31;13:1784. doi: 10.1038/s41598-022-26962-w (PMC9889352; doi:10.1038/s41598-022-26962-w)
Supplement: Supplementary file 4 — Supplementary Information 4. [file 41598_2022_26962_MOESM4_ESM.docx]

# **Supplemental A: Diagnostic Codes and the Search Strings**

| **Diagnosis Code** | **Search Test** |
| --- | --- |
| dx02 | "adenocarcinoma" |
| dx03 | "squamous carcinoma" |
| dx03 | "squamous cell carcinoma" |
| dx04 | "small cell carcinoma" |
| dx04 | "small cell neuroendocrine carcinoma" |
| dx05 | "non-small cell carcinoma" |
| dx05 | "non small cell carcinoma" |
| dx05 | "nonsmall cell carcinoma" |
| dx06 | "large cell neuroendocrine carcinoma" |
| dx07 | "atypical carcinoid" |
| dx08 | "typical carcinoid" |
| dx08 | "carcinoid" |
| dx08 | "neuroendocrine tumor" |
| dx09 | "Atypical mesothelial hyperplasia" |
| dx10 | "mesothelioma" |
| dx15 | "Atypical glandular proliferation" |
| dx15 | "Atypical adenomatous hyperplasia" |
| dx15 | "atypical alveolar hyperplasia" |
| dx15 | "atypical alveolar cells" |
| dx15 | "atypical bronchioalveolar proliferation" |
| dx20 | "malignant" |
| dx20 | "malignant tumor" |
| dx20 | "malignant tumour" |
| dx20 | "malignant neoplasm" |
| dx20 | "consistent with malignancy" |
| dx20 | "Poorly differentiated neoplasm" |
| dx20 | "Poorly differentiated malignancy" |
| dx20 | "Malignant epithelial neoplasm" |
| dx20 | "MALIGNANT GLANDS SEEN” |
| dx20 | "Malignant glandular neoplasm" |
| dx20 | "Myxoid neoplasm" |
| dx20 | "Mucinous neoplasm" |
| dx20 | "Invasive Epitheloid neoplasm" |
| dx20 | "Positive for epithelial neoplasm" |
| dx21 | "LYMPHOPROLIFERATIVE DISORDER" |
| dx21 | "lymphoproliferative process" |
| dx21 | "monotonous lymphoid infiltrate" |
| dx21 | "Atypical lymphoid aggregate" |
| dx21 | "Atypical lymphoid infiltrate" |
| dx21 | "suggestive of lymphocytic proliferation" |
| dx21 | "suspicious for lymphoplasmacytic proliferative disorder" |
| dx21 | "Dense lymphocytic infiltrative proliferation" |
| dx22 | "lymphoma" |
| dx23 | "sarcoma " |
| dx23 | "sarcoma," |
| dx25 | "Soft tissue neoplasm" |
| dx25 | "spindle cell lesion" |
| dx25 | "Spindle cell neoplasm" |
| dx27 | "suspect for neoplasm" |
| dx27 | "atypical cells identified" |
| dx27 | "atypical cells present" |
| dx27 | "Few atypical cells" |
| dx27 | "atypical but degenerated cells" |
| dx27 | "atypical, but degenerated cells" |
| dx27 | "atypical degenerated group of cells " |
| dx27 | "atypical epithelioid cells" |
| dx27 | "Atypical epithelial cells" |
| dx27 | "Atypical alveolar epithelial cells" |
| dx27 | "atypical glands" |
| dx27 | "atypical cell" |
| dx27 | "atypical single cells" |
| dx27 | "Atypical small cells" |
| dx27 | "atypical small/medium epithelial cells" |
| dx27 | "Atypical infiltrate" |
| dx27 | "Atypical glandular epithelium" |
| dx27 | "Atypical glandular cells" |
| dx27 | "Atypical respiratory cells" |
| dx27 | "Atypical bronchial cells" |
| dx27 | "Atypical squamous cell" |
| dx27 | "atypical keratinized squamous cells" |
| dx27 | "Atypical squamous epithelial" |
| dx27 | "Atypical squamous epithelium" |
| dx27 | "atypical squamoid cells" |
| dx27 | "atypical squamous metaplastic cells" |
| dx27 | "atypical bronchioalveolar lining cells" |
| dx27 | "atypical group of cells" |
| dx27 | "atypical large epithelial cells" |
| dx27 | "atypical scanty material with cells" |
| dx27 | "minimal cytologic atypia" |
| dx27 | "abnormal cells" |
| dx27 | "suspicious for a neoplasm" |
| dx27 | "suspicious for malignancy" |
| dx27 | "suspect for malignancy" |
| dx27 | "suggestive of malignancy" |
| dx27 | "cannot rule out malignancy" |
| dx27 | "Cannot exclude malignancy" |
| dx27 | "malignancy cannot be excluded" |
| dx27 | "cannot confidently exclude malignancy" |
| dx27 | "malignancy not excluded" |
| dx28 | "Suspicious for a Non-Hodgkin's lymphoma" |
| dx28 | "Suspicious for non-small cell carcinoma" |
| dx28 | "Suspicious for non small cell carcinoma" |
| dx28 | "Suspicious for nonsmall cell carcinoma" |
| dx28 | "Suspicious for poorly differentiated carcinoma" |
| dx28 | "Suspicious for small cell carcinoma" |
| dx28 | "Suspicious for adenocarcinoma" |
| dx28 | "Suspicious for squamous cell carcinoma" |
| dx28 | "Suspicious for squamous carcinoma" |
| dx28 | "Suspicious for lymphoma" |
| dx28 | "Suspicious for carcinoma" |
| dx29 | "pulmonary adenocarcinoma" |
| dx29 | "lung primary" |
| dx30 | "metastatic" |
| dx30 | "metastasis" |
| dx31 | "colon" |
| dx31 | "rectal" |
| dx31 | "colorectal" |
| dx32 | "breast" |
| dx33 | "urothelial carcinoma" |
| dx33 | "urothelial cell carcinoma" |
| dx33 | "transitional cell carcinoma" |
| dx33 | "bladder" |
| dx34 | "prostatic origin" |
| dx34 | "prostate" |
| dx35 | "thyroid" |
| dx36 | "melanoma" |
| dx37 | "kidney" |
| dx37 | "renal" |
| dx40 | "thymoma" |
| dx41 | "sclerosing hemangioma" |
| dx41 | "pneumocytoma" |
| dx50 | "carcinoma" |
| dx60 | "necrosis" |
| dx60 | "necrotic" |
| dx60 | "necrotizing inflammation" |
| dx61 | "respiratory bronchiolitis interstitial lung disease" |
| dx61 | "RBILD" |
| dx61 | "smoker's respiratory bronchiolitis" |
| dx61 | "Features of respiratory bronchiolitis" |
| dx65 | "granulation tissue" |
| dx66 | "squamous papilloma" |
| dx67 | "vegetable matter" |
| dx70 | "Benign." |
| dx70 | "Benign Cellular aspirate" |
| dx70 | "Benign Moderately cellular aspirate" |
| dx70 | "favour benign" |
| dx70 | "favor benign" |
| dx70 | "favour reactive" |
| dx70 | "favor reactive" |
| dx70 | "Benign lymph node" |
| dx70 | "Benign anthracotic lymph node" |
| dx70 | "Benign, scanty aspirate" |
| dx70 | "Scantly cellular aspirate consisting of occasional lymphocytes" |
| dx70 | "Reactive ciliated respiratory and reserve cells present." |
| dx70 | "Benign-appearing bronchial tissue" |
| dx70 | "Benign bronchial tissue" |
| dx70 | "unremarkable bronchial mucosa" |
| dx70 | "Benign bronchial mucosa" |
| dx70 | "benign bronchial epithelium" |
| dx70 | "Mainly bronchial cells present" |
| dx70 | "Bronchial mucosa with no pathologic finding" |
| dx70 | "Benign-appearing respiratory mucosa" |
| dx70 | "Benign respiratory mucosa" |
| dx70 | "unremarkable endobronchial tissue" |
| dx70 | "Unremarkable endobronchial mucosa" |
| dx70 | "Unremarkable bronchial wall tissue " |
| dx70 | "Unremarkable tiny fragments of endobronchial mucosa" |
| dx70 | "BENIGN RESPIRATORY EPITHELIUM" |
| dx70 | "respiratory epithelium without significant diagnostic abnormality" |
| dx70 | "Unremarkable fragments of cartilage and endobronchial mucosa" |
| dx70 | "UNREMARKABLE CARTILAGE AND BRONCHIAL TISSUE" |
| dx70 | "Benign bronchial wall" |
| dx70 | "Bronchial mucosa without findings" |
| dx70 | "Benign chronic inflamed respiratory mucosa" |
| dx70 | "Benign, chronic inflamed bronchial mucosa" |
| dx70 | "Benign edematous bronchial tissue" |
| dx70 | "Edematous bronchial mucosa with no other specific pathology" |
| dx70 | "benign pulmonary parenchyma" |
| dx70 | "unremarkable lung parenchyma" |
| dx70 | "Benign lung tissue" |
| dx70 | "Benign alveolar lung tissue" |
| dx70 | "Benign alveolar lung parenchyma " |
| dx70 | "Benign-appearing lung parenchyma" |
| dx70 | "Benign lung parenchyma" |
| dx70 | "Benign parenchymal lung tissue" |
| dx70 | "Lung parenchymal tissue with no significant histological abnormality" |
| dx70 | "Bronchial and lung alveolar tissue without specific diagnostic abnormality" |
| dx70 | "Benign bronchial and alveolar tissue" |
| dx70 | "Benign bronchial and alveolar lung tissue" |
| dx70 | "Benign parenchymal lung" |
| dx70 | "benign lung tissue" |
| dx70 | "Benign lung and bronchial parenchyma" |
| dx70 | "Alveolar tissue with no significant findings" |
| dx70 | "Alveolar lung tissue without diagnostic abnormality" |
| dx70 | "very small amount of bland epithelium" |
| dx70 | "BENIGN FIBROADIPOSE TISSUE AND BLOOD" |
| dx70 | "Alveolar tissue without significant findings" |
| dx70 | "Mild chronic inflammation, no specific findings" |
| dx70 | "Minimal chronic inflammation, no other findings" |
| dx70 | "Reactive bronchial cells" |
| dx70 | "bronchial cells present in a background of lymphocytes" |
| dx70 | "lymphoid material with rare anthracotic-laden macrophages" |
| dx70 | "benign ciliated respiratory cells only" |
| dx71 | "Organized pneumonia" |
| dx71 | "Organizing pneumonia" |
| dx71 | "Bronchopneumonia with focal organization" |
| dx72 | "granuloma" |
| dx72 | "GRANULOMMATOUS INFLAMMATION" |
| dx72 | "epithelioid histiocytes" |
| dx72 | "suggestive of granulommatous process" |
| dx72 | "likely a granulommatous process" |
| dx73 | "Nonspecific scar" |
| dx73 | "suggestive of scar" |
| dx73 | "Scarred lung tissue" |
| dx73 | "Scarred alveolar lung" |
| dx73 | "scarlike tissue" |
| dx73 | "Scarring" |
| dx73 | "Scarred lung" |
| dx73 | "scar tissue " |
| dx73 | "Dense scar" |
| dx73 | "Fibroelastotic scar" |
| dx73 | "Bland fibrosis" |
| dx74 | "Hamartoma" |
| dx75 | "solitary (isolate) fibrous tumour" |
| dx75 | "solitary fibrous tumour" |
| dx75 | "solitary fibrous tumor" |
| dx75 | "solitary/localized fibrous tumor" |
| dx76 | "pleural plaque" |
| dx77 | "Langerhans Cell Histiocytosis" |
| dx78 | "Amyloid" |
| dx79 | "Mixed population of lymphocytes" |
| dx79 | "Benign lymphocytes" |
| dx79 | "Mainly lymphocytes noted" |
| dx79 | "Benign, consistent with lymph node" |
| dx79 | "benign mixed lymphocytes" |
| dx79 | "benign aspirate consisting of occasional lymphocytes" |
| dx79 | "Normal lymph node elements identified" |
| dx79 | "benign lymphoid material present" |
| dx79 | "benign lymph nodal tissue" |
| dx79 | "Only scant lymphocytes noted" |
| dx79 | "Benign, reactive lymphoid proliferation" |
| dx79 | "Lymphocytic infiltrate, favor reactive or inflammatory process" |
| dx79 | "Lymphocytic infiltrate, favour benign" |
| dx79 | "Bronchial mucosa showing mild acute and chronic inflammation" |
| dx79 | "Bronchial mucosa with mild chronic inflammation and some cartilage" |
| dx79 | "Bronchial mucosa with mild chronic inflammation" |
| dx79 | "Endobronchial mucosa with mild chronic inflammation" |
| dx79 | "lung alveolar tissue showing mild chronic inflammation" |
| dx79 | "Bronchial tissue showing chronic inflammation" |
| dx79 | "Bronchial tissue showing mild chronic inflammation" |
| dx79 | "Respiratory mucosa with mild chronic inflammation" |
| dx79 | "Lung parenchyma with fragment of cartilage and calcification" |
| dx79 | "Benign-appearing fibroadipose tissue" |
| dx79 | "Bronchial mucosa with eosinophilia" |
| dx79 | "Mainly blood with lymphocytes" |
| dx79 | "lymphocytes intermixed with bronchial cells" |
| dx79 | "mainly benign lymphoid material" |
| dx79 | "Mainly lymphocytes are present" |
| dx79 | "consists of lymphoid material" |
| dx79 | "Bronchial cells present in a background of lymphocytes" |
| dx79 | "Benign appearing lymphocytes" |
| dx79 | "Polymorphous lymphocytes" |
| dx79 | "mixed population of few lymphocytes present" |
| dx79 | "Scant lymphocytes present" |
| dx79 | "scant mixed lymphocytes noted" |
| dx80 | "No diagnostic abnormality" |
| dx80 | "No significant pathology" |
| dx80 | "NO DEFINITE PATHOLOGICAL DIAGNOSIS" |
| dx80 | "no histopathological abnormality" |
| dx80 | "no significant abnormality" |
| dx80 | "No significant findings" |
| dx80 | "No findings" |
| dx80 | "no significant pathological abnormality" |
| dx80 | "No Significant Pathological Changes" |
| dx80 | "no significant histological abnormality" |
| dx80 | "No pathologic diagnosis" |
| dx80 | "no apparent lesional tissue" |
| dx80 | "no lesional tissue" |
| dx80 | "without significant pathology" |
| dx80 | "negative for significant pathology" |
| dx80 | "without significant histopathologic abnormality" |
| dx90 | "Negative for malignancy" |
| dx90 | "NEGATIVE for evidence of malignancy" |
| dx90 | "Negative for malignant cells" |
| dx90 | "No tumor or malignancy" |
| dx90 | "No obvious malignant cells" |
| dx90 | "No definitive malignant cells" |
| dx90 | "No overtly malignant cells" |
| dx90 | "No clearly malignant cells" |
| dx90 | "No viable malignant cells" |
| dx90 | "No obviously malignant cells" |
| dx90 | "No malignant cells" |
| dx90 | "No malignancy identified" |
| dx90 | "No malignancy in these biopsies" |
| dx90 | "No malignancy is seen" |
| dx90 | "NO EVIDENCE OF MALIGNANCY" |
| dx90 | "No evidence of dysplasia and malignancy" |
| dx90 | "No definite evidence of malignancy" |
| dx90 | "No malignancy is identified" |
| dx90 | "No evidence of dysplasia or malignancy" |
| dx90 | "Negative for dysplasia or malignancy" |
| dx90 | "Negative for dysplasia and malignancy" |
| dx90 | "no evidence of inflammation, dysplasia or malignancy" |
| dx90 | "Negative for epithelial malignancy" |
| dx90 | "Negative for carcinoma" |
| dx91 | "no obvious epithelial neoplasm identified" |
| dx91 | "negative for lung tissue or neoplasm" |
| dx91 | "negative for neoplasia" |
| dx91 | "No evidence of neoplasia" |
| dx91 | "no evidence of a tumour" |
| dx91 | "No tumor present" |
| dx91 | "No tumor identified" |
| dx91 | "No tumors identified" |
| dx91 | "Negative for neoplasm" |
| dx91 | "No neoplastic tissue" |
| dx91 | "No evidence of viable neoplasia" |
| dx91 | "Negative for primary or metastatic malignancy" |
| dx92 | "No evidence of metastatic malignancy" |
| dx92 | "No evidence of metastatic disease" |
| dx92 | "Negative for metastatic malignancy" |
| dx92 | "No metastatic disease" |
| dx92 | "no evidence of metastatic malignancy" |
| dx92 | "no evidence of metastasis" |
| dx92 | "Negative for metastasis" |
| dx92 | "Negative for metastatic carcinoma" |
| dx93 | "No evidence of metastatic or granulomatous disease" |
| dx93 | "No evidence of granulomatous disease or metastatic malignancy" |
| dx93 | "Negative for granulomatous disease or metastatic malignancy" |
| dx93 | "Negative for metastatic malignancy or granulomatous disease" |
| dx95 | "likely not representative" |
| dx95 | "may not be representative" |
| dx95 | "may not be a representative tissue" |
| dx95 | "not representative of the lesion" |
| dx95 | "Clinical correlation required regarding representation" |
| dx95 | "? representative" |
| dx95 | "not a representative sample" |
| dx95 | "Suboptimal specimen" |
| dx95 | "Suboptimal for cytological assessment" |
| dx95 | "Sub-optimal for cytologic interpretation" |
| dx99 | "Insufficient lymphoid material" |
| dx99 | "Insufficient lymphoid tissue for cytological interpretation" |
| dx99 | "Insufficient lymphoid material for cytological interpretation" |
| dx99 | "Insufficient nodal tissue present" |
| dx99 | "Insufficient for evaluation" |
| dx99 | "Inadequate for cytological interpretation" |
| dx99 | "Insufficient for cytological interpretation" |
| dx99 | "Unsatisfactory material for cytological interpretation" |
| dx99 | "Unsatisfactory for proper evaluation" |
| dx99 | "Unsatisfactory due to insufficient cellularity" |
| dx99 | "Insufficient due to inadequate cellularity" |
| dx99 | "Insufficient due to scant cellularity" |
| dx99 | "Insufficient due to low cellularity" |
| dx99 | "Insufficient cellular material for proper assessment" |
| dx99 | "Specimen is insufficient for assessment" |
| dx99 | "Unsatisfactory because of inadequate cellularity" |
| dx99 | "Unsatisfactory for examination" |
| dx99 | "Inadequate material for cytological interpretation" |
| dx99 | "may not be a representative sample" |
| dx99 | "unsatisfactory sample" |
| dx99 | "Insufficient specimen for cytological evaluation" |
| dx99 | "Insufficient for cytological evaluation" |
| dx99 | "Unsatisfactory for cytological evaluation" |
| dx99 | "May not be a representative sample" |
| dx99 | "not specific" |
| dx99 | "no specific diagnosis" |
| dx99 | "Non-diagnostic" |
| dx99 | "Non diagnostic" |
| dx99 | "not diagnostic" |
| dx99 | "nondiagnostic" |
| dx99 | "Not a diagnostic specimen" |
| dx99 | "Unsatisfactory." |
| dx99 | "Unsatisfactory specimen" |
| dx99 | "Unsatisfactory for cytological assessment" |
| dx99 | "Unsatisfactory for cytological interpretation" |
| dx99 | "Unsatisfactory for cytologic assessment" |
| dx99 | "Unsatisfactory due to scant cellularity" |
| dx99 | "not further diagnostic" |
| dx99 | "MARKEDLY DEGENERATE SAMPLE" |
| dx99 | "Material not assessable" |
| dx99 | "Insufficient tissue for pathologic evaluation" |
| dx99 | "No diagnostic material" |
| dx99 | "no diagnostic lung tissue" |
| dx99 | "No viable kidney tissue present" |
| dx99 | "crushed urothelial tissue" |
| dx99 | "Scanty crushed tissue" |
| dx99 | "insufficient for diagnosis" |
| dx99 | "Insufficient tissue for diagnosis" |
| dx99 | "Material insufficient" |
| dx99 | "INSUFFICIENT TISSUE FOR ASSESSMENT" |
| dx99 | "insufficient material" |
| dx99 | "Not a sufficient sample" |
| dx99 | "Inconclusive" |
| dx99 | "Tissue insufficient" |
| dx99 | "Tissue did not survive the processing" |
| dx99 | "No Tissue Material" |
| dx99 | "No tissue is seen at microscopy" |
| dx99 | "Material insufficient for assessment" |

# **Supplemental B: Location Codes and the Search Strings**

| **Location Code** | **Search Text** |
| --- | --- |
| ln_st1r | ' 1R' |
| ln_st2r | ' 2R' |
| ln_st2r | 'LYMPH NODE R2' |
| ln_st2l | ' 2L' |
| ln_st2p | ' 2P' |
| ln_st3 | 'LYMPH NODE 3 ' |
| ln_st3p | ' 3P' |
| ln_st4 | 'LYMPH NODE 4 ' |
| ln_st4 | 'LYMPH NODE 4C' |
| ln_st4 | 'LYMPH NODE - 4 C' |
| ln_st4r | ' 4R' |
| ln_st4r | 'LYMPH NODE R4' |
| ln_st4r | 'LYMPH NODE - 4 R' |
| ln_st4r | '4R LYMPH NODE' |
| ln_st4r | 'LYMPH NODE -4R' |
| ln_st4l | ' 4L' |
| ln_st4l | 'LYMPH NODE - 4 L' |
| ln_st5 | 'LYMPH NODE 5' |
| ln_st6 | 'LYMPH NODE - 6' |
| ln_st7 | 'LYMPH NODE 7' |
| ln_st7 | 'LYMPH NODE #7' |
| ln_st7 | 'LYMPH NODE - 7' |
| ln_st7 | 'LYMPH NODES - 7' |
| ln_st7 | 'LYMPH NODE - #7' |
| ln_st7 | 'SUBCARINAL' |
| ln_st7 | '7 LYMPH NODE' |
| ln_st7 | 'STATION 7 LYMPH NODE' |
| ln_st8 | 'LYMPH NODE 8 ' |
| ln_st8 | '8 LYMPH NODE' |
| ln_st8r | 'LYMPH NODE - 8R' |
| ln_st8r | 'LYMPH NODE 8R' |
| ln_st8l | 'LYMPH NODES - 8L' |
| ln_st8l | 'LYMPH NODES 8L' |
| ln_st9 | 'LYMPH NODE 9' |
| ln_st10r | ' 10R' |
| ln_st10r | '10 R' |
| ln_st10l | ' 10L' |
| ln_st10l | ' 10 L' |
| ln_st11r | ' 11R' |
| ln_st11r | ' 11 R' |
| ln_st11l | ' 11L' |
| ln_st11l | ' 11 L' |
| ln_st12r | ' 12R' |
| ln_st12r | ' 12 R' |
| ln_st12l | ' 12L' |
| ln_st12l | ' 12 L' |
| ln_celiac | 'CELIAC NODE' |
| ln_celiac | 'CELIAC LYMPH NODE' |
| ln_gashep | 'GASTROHEPATIC LYMPH' |
| ln_gashep | 'LYMPH NODE - EUS GASTROHEPATIC' |

**Supplemental C: Mutually Exclusive Group and Diagnosis Codes**

| **Mutually Exclusive Group** | **Diagnosis Codes** |
| --- | --- |
| Malignant | dx02, dx03, dx04, dx05, dx06, dx10, dx20, dx22, dx23, dx30, dx36, dx50 |
| Suspicious | dx21, dx27, dx28 |
| Insufficient | dx95, dx99 |
| Benign | dx60, dx61, dx65, dx66, dx67, dx70, dx71, dx72, dx73, dx74, dx75, dx76, dx77, dx78, dx79, dx80, dx90, dx91, dx92, dx93 |
