# Supplementary material for: The physician factor and anatomical site in 8846 consecutive mediastinal lymph node aspirations in a cross-sectional study
Source: Sci Rep. 2023 Jan 31;13:1784. doi: 10.1038/s41598-022-26962-w (PMC9889352; doi:10.1038/s41598-022-26962-w)
Supplement: Supplementary file 5 — Supplementary Information 5. [file 41598_2022_26962_MOESM5_ESM.pdf]

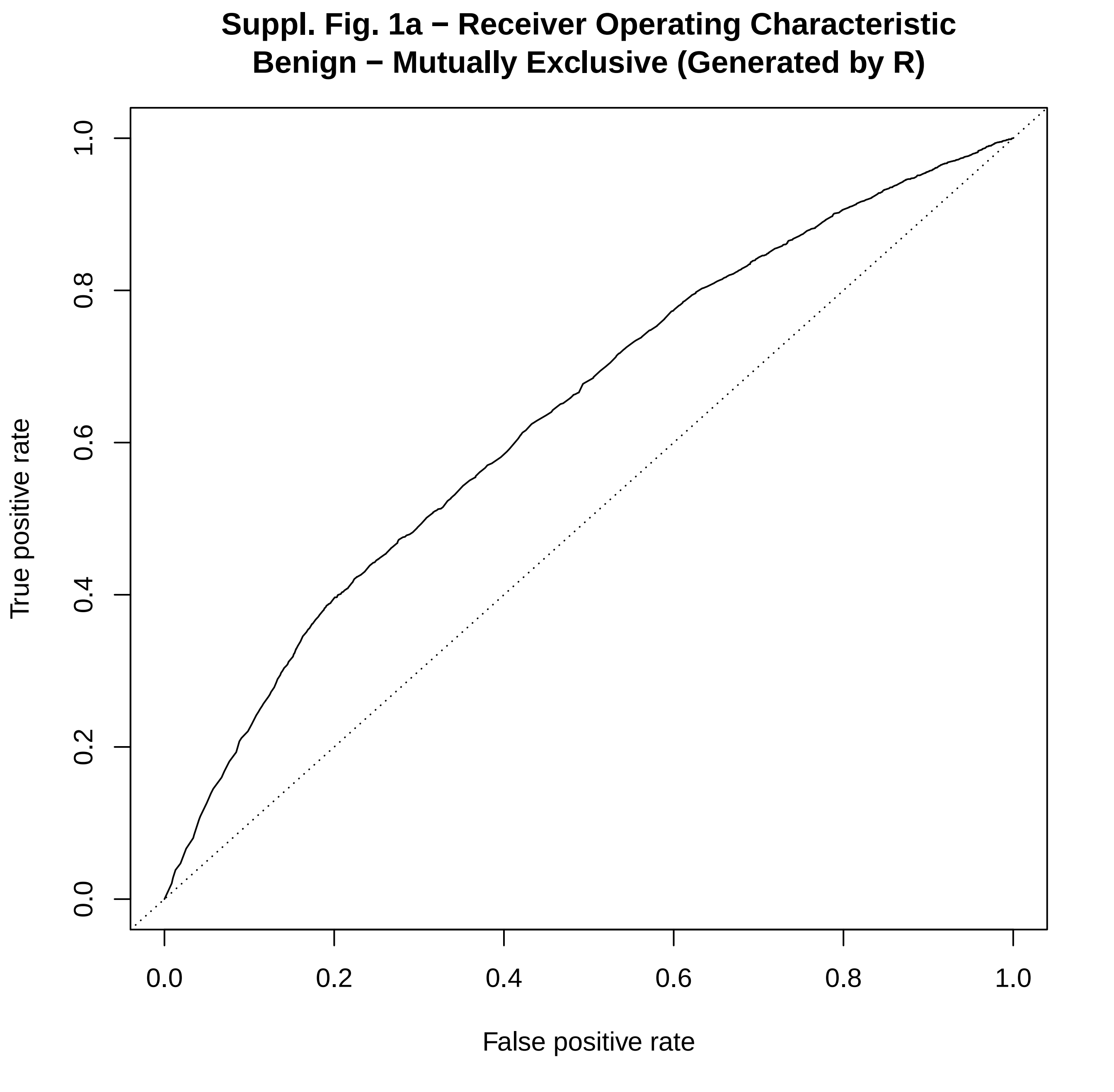

Suppl. Fig. 1a − Receiver Operating Characteristic
Benign − Mutually Exclusive (Generated by R)
True positive rate
0.0
0.2
0.4
0.6
0.8
1.0
False positive rate

Suppl. Fig. 1b − Receiver Operating Characteristic
Benign − Mutually Exclusive (Generated by R)

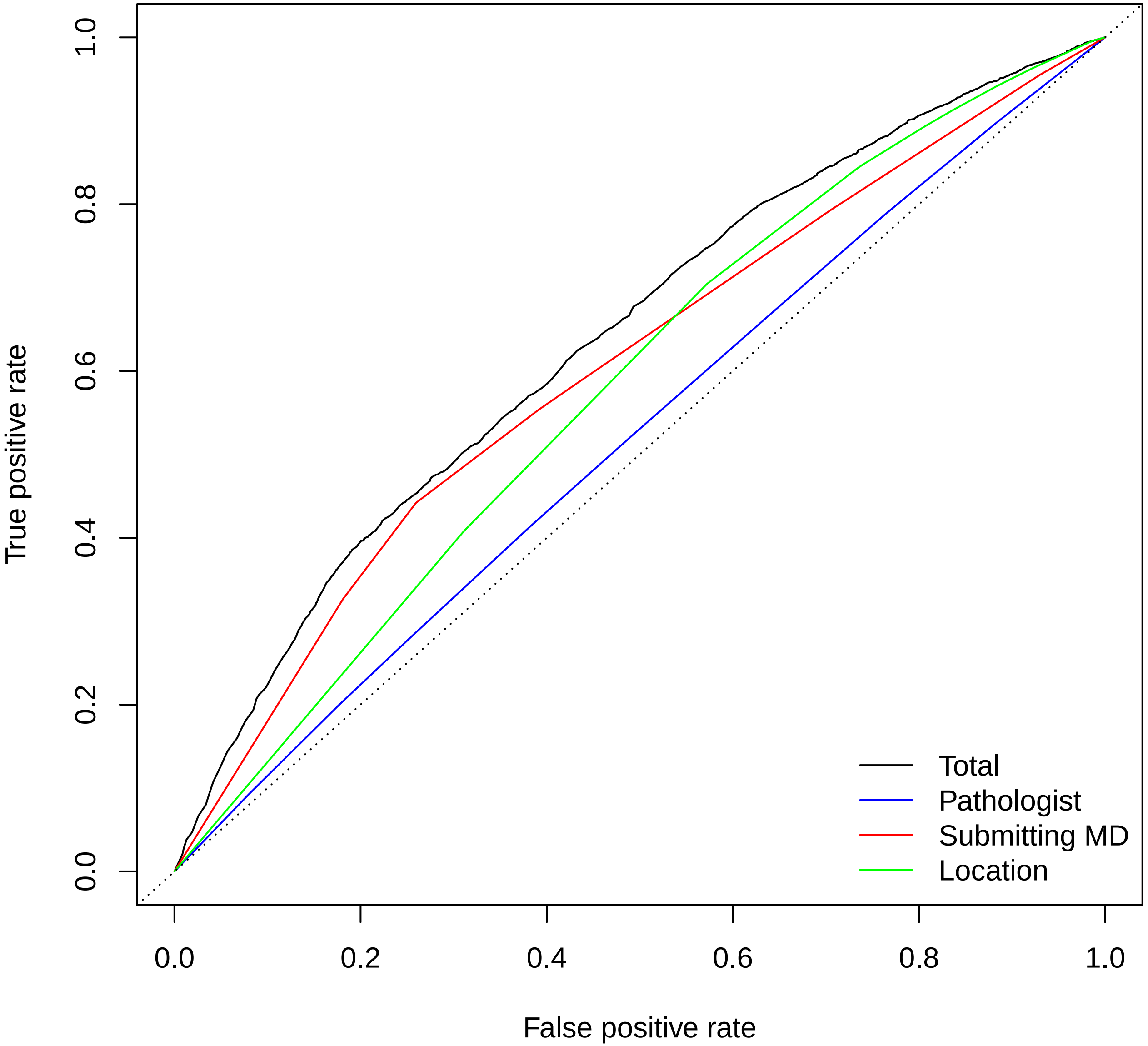

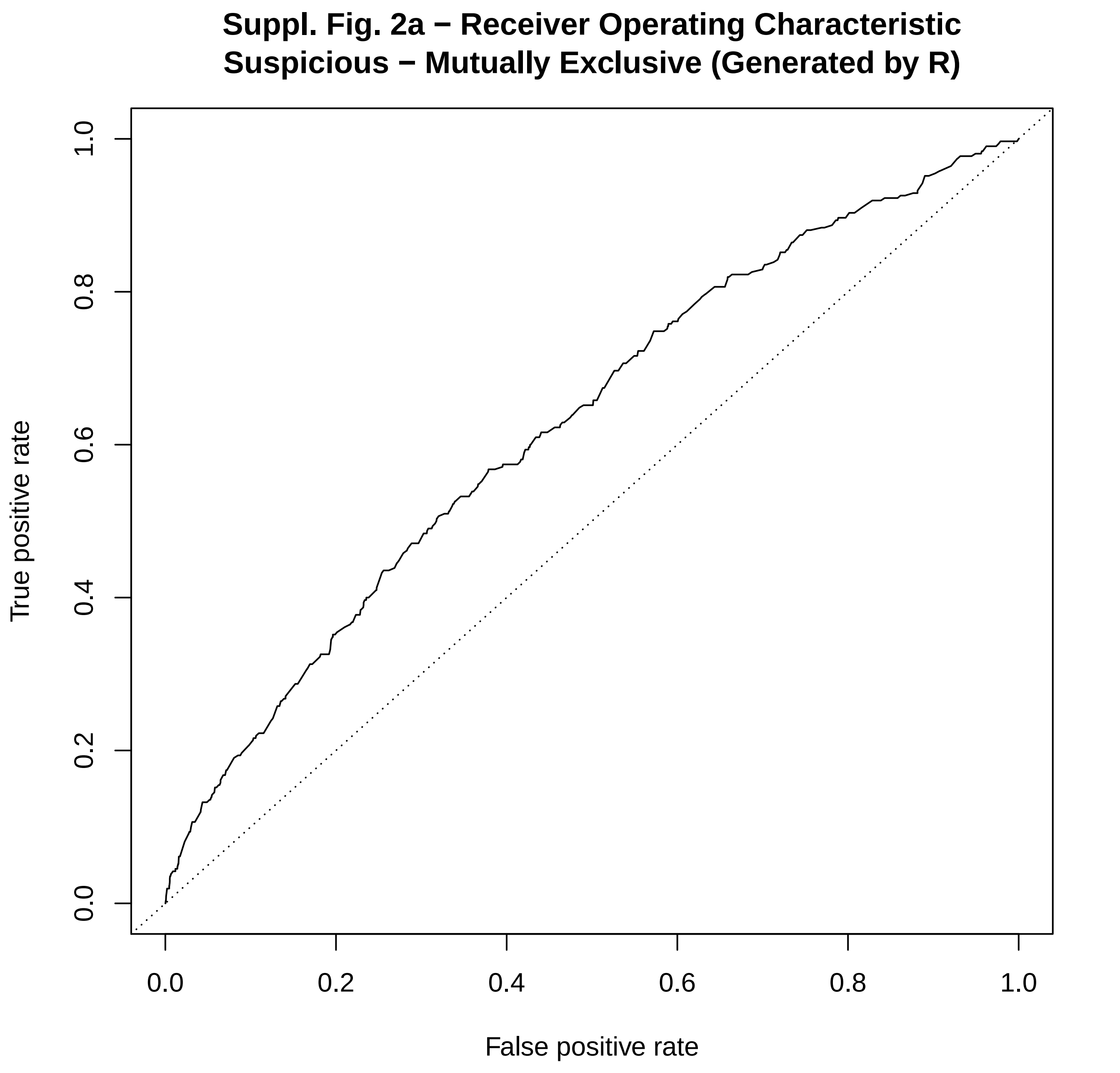
Suppl. Fig. 2a − Receiver Operating Characteristic
Suspicious − Mutually Exclusive (Generated by R)
True positive rate
False positive rate
0.0
0.2
0.4
0.6
0.8
1.0

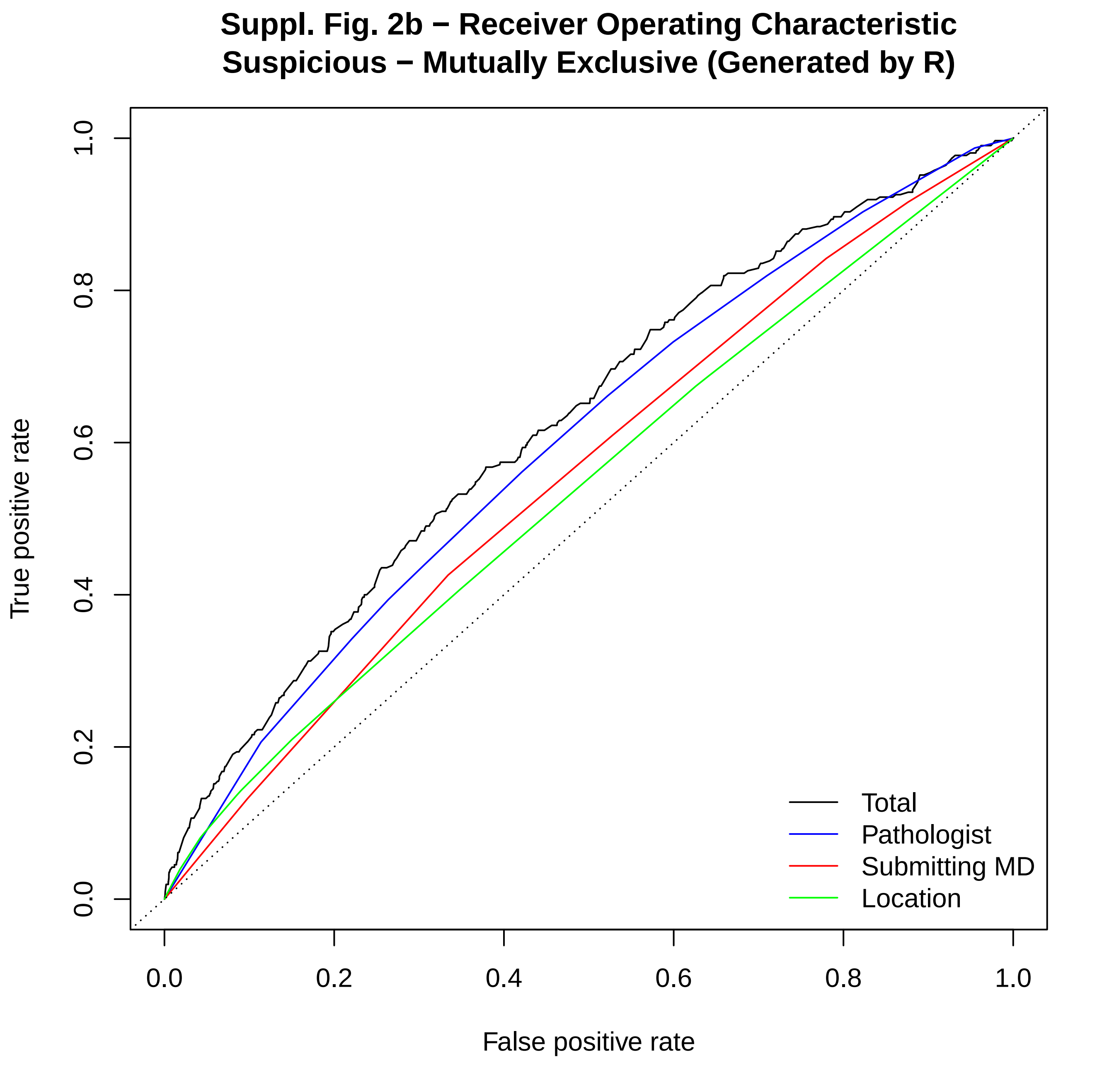
Suppl. Fig. 2b − Receiver Operating Characteristic
Suspicious − Mutually Exclusive (Generated by R)
True positive rate
False positive rate
0.0
0.2
0.4
0.6
0.8
1.0
Total
Pathologist
Submitting MD
Location

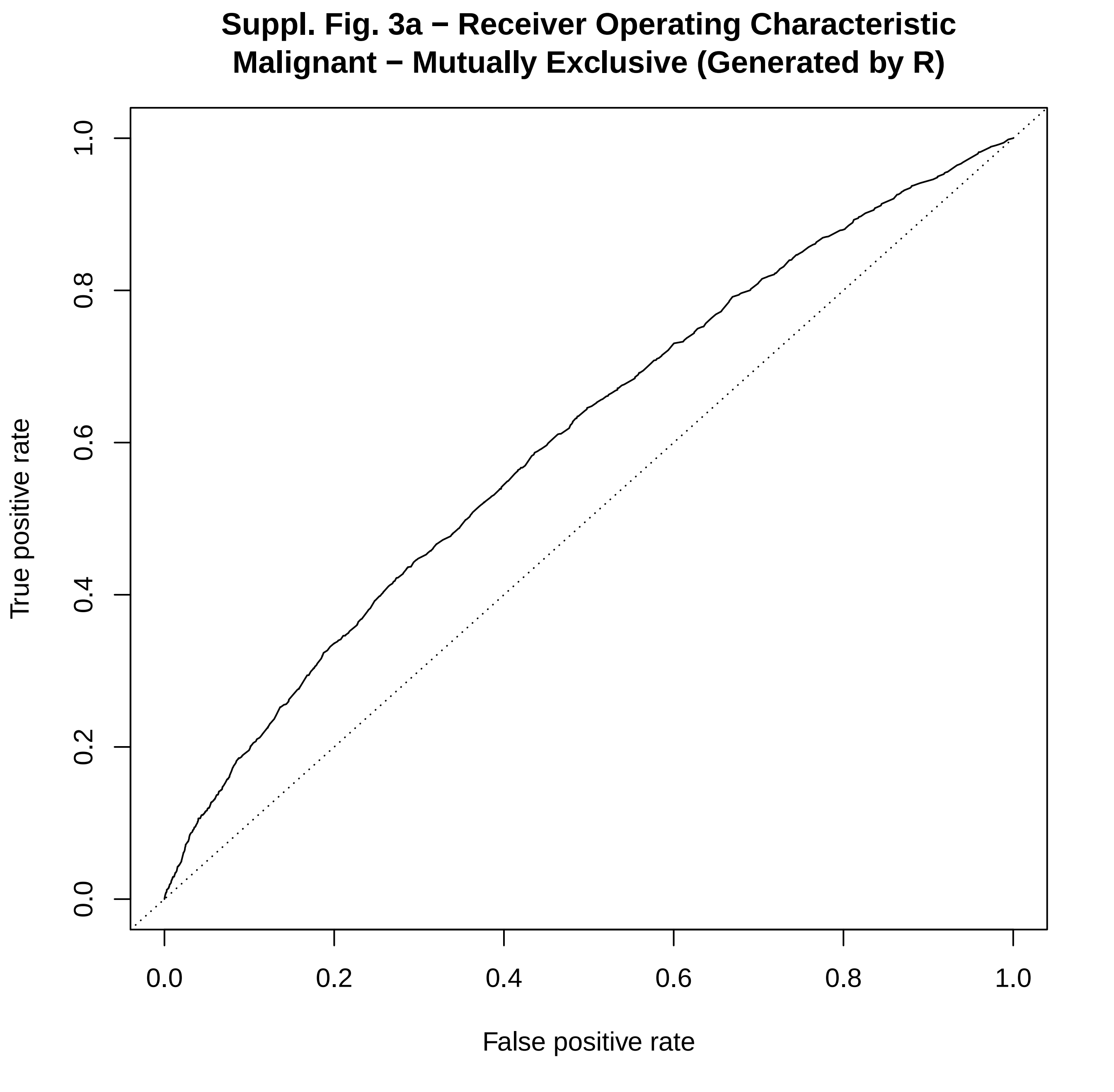
Suppl. Fig. 3a − Receiver Operating Characteristic
Malignant − Mutually Exclusive (Generated by R)
True positive rate
0.0
0.2
0.4
0.6
0.8
1.0
False positive rate
0.0
0.2
0.4
0.6
0.8
1.0

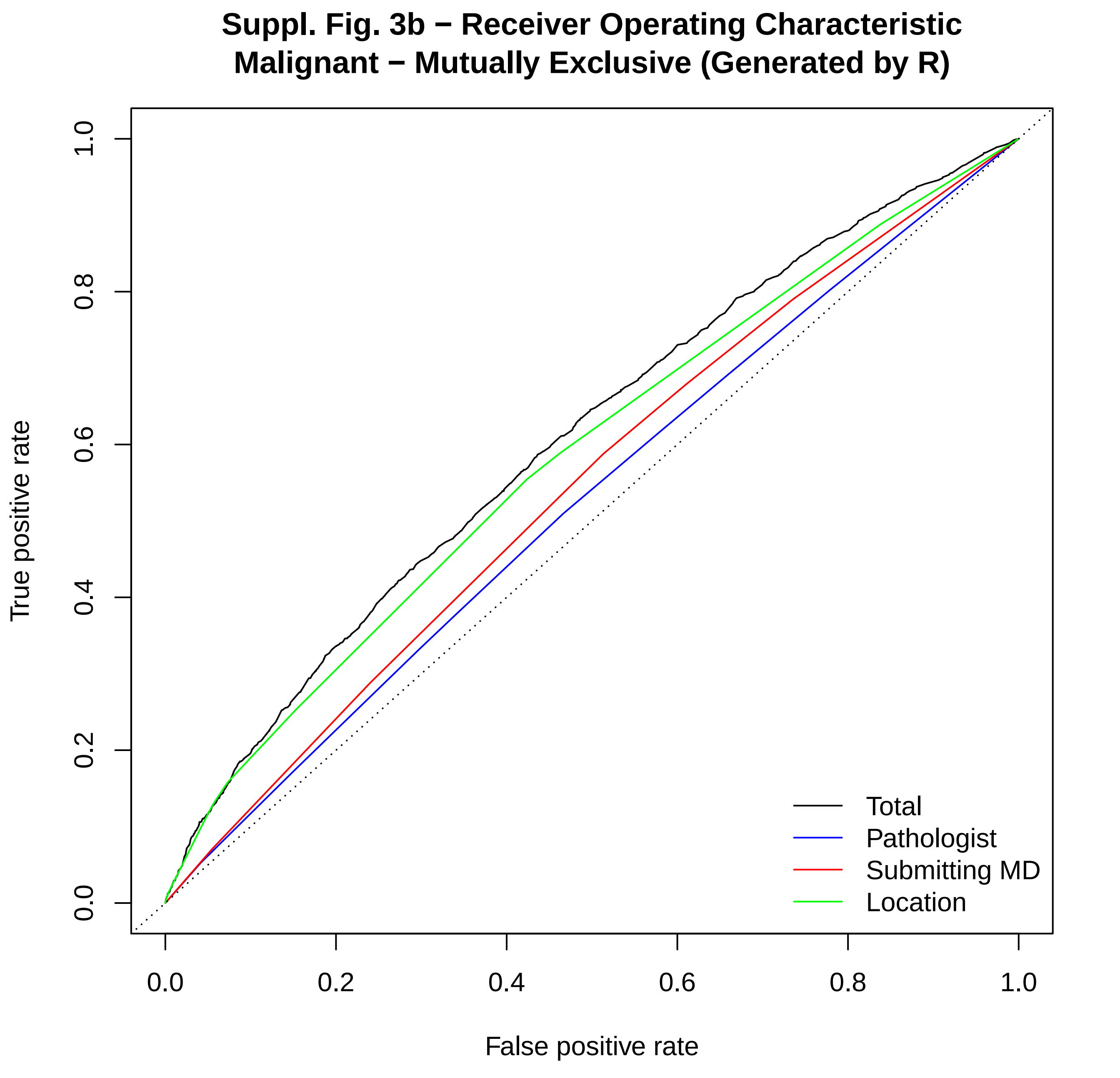
Suppl. Fig. 3b − Receiver Operating Characteristic
Malignant − Mutually Exclusive (Generated by R)
True positive rate
False positive rate
0.0
0.2
0.4
0.6
0.8
1.0
Total
Pathologist
Submitting MD
Location

Suppl. Fig. 4a − Receiver Operating Characteristic
Insufficient − Mutually Exclusive (Generated by R)

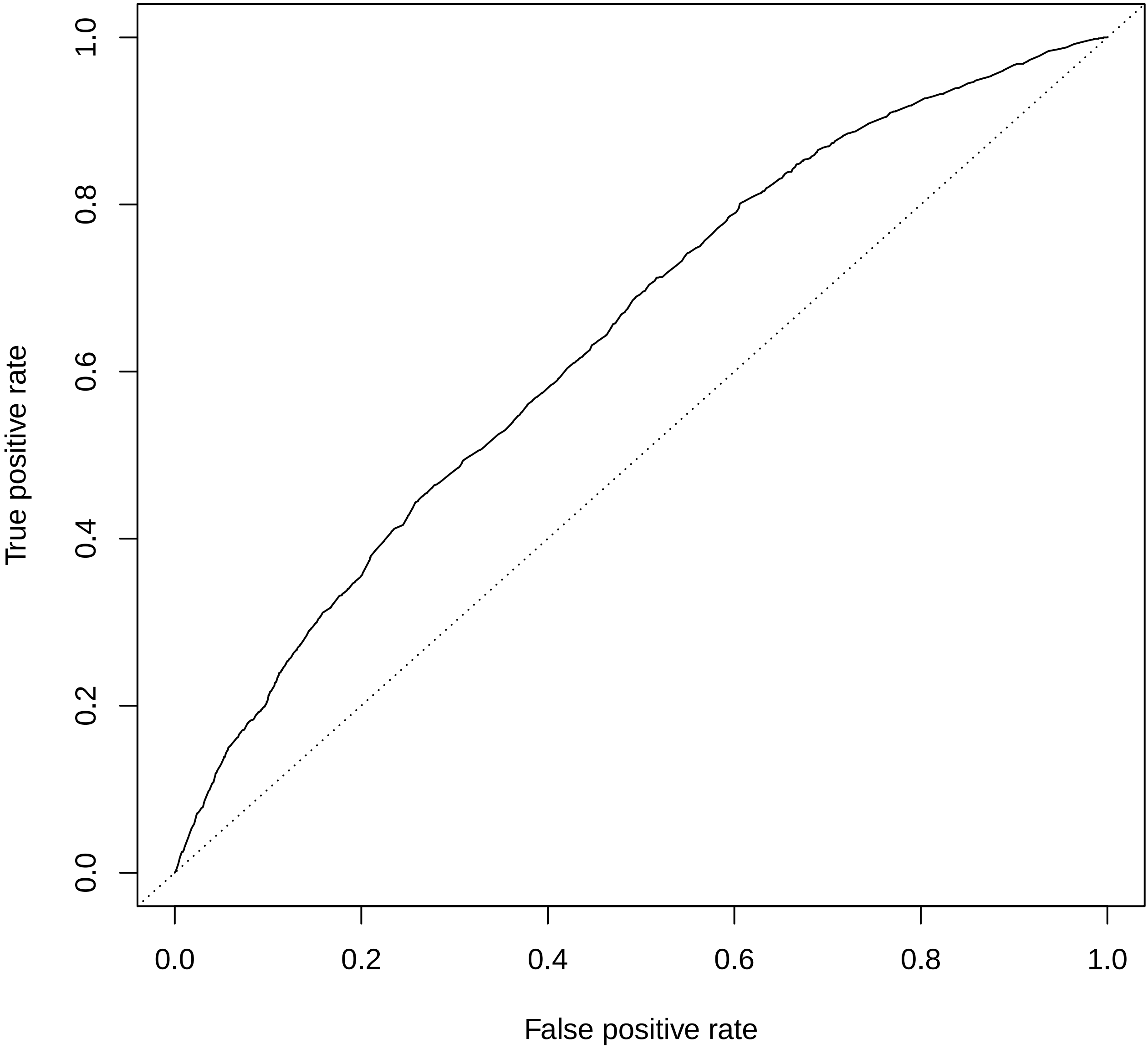

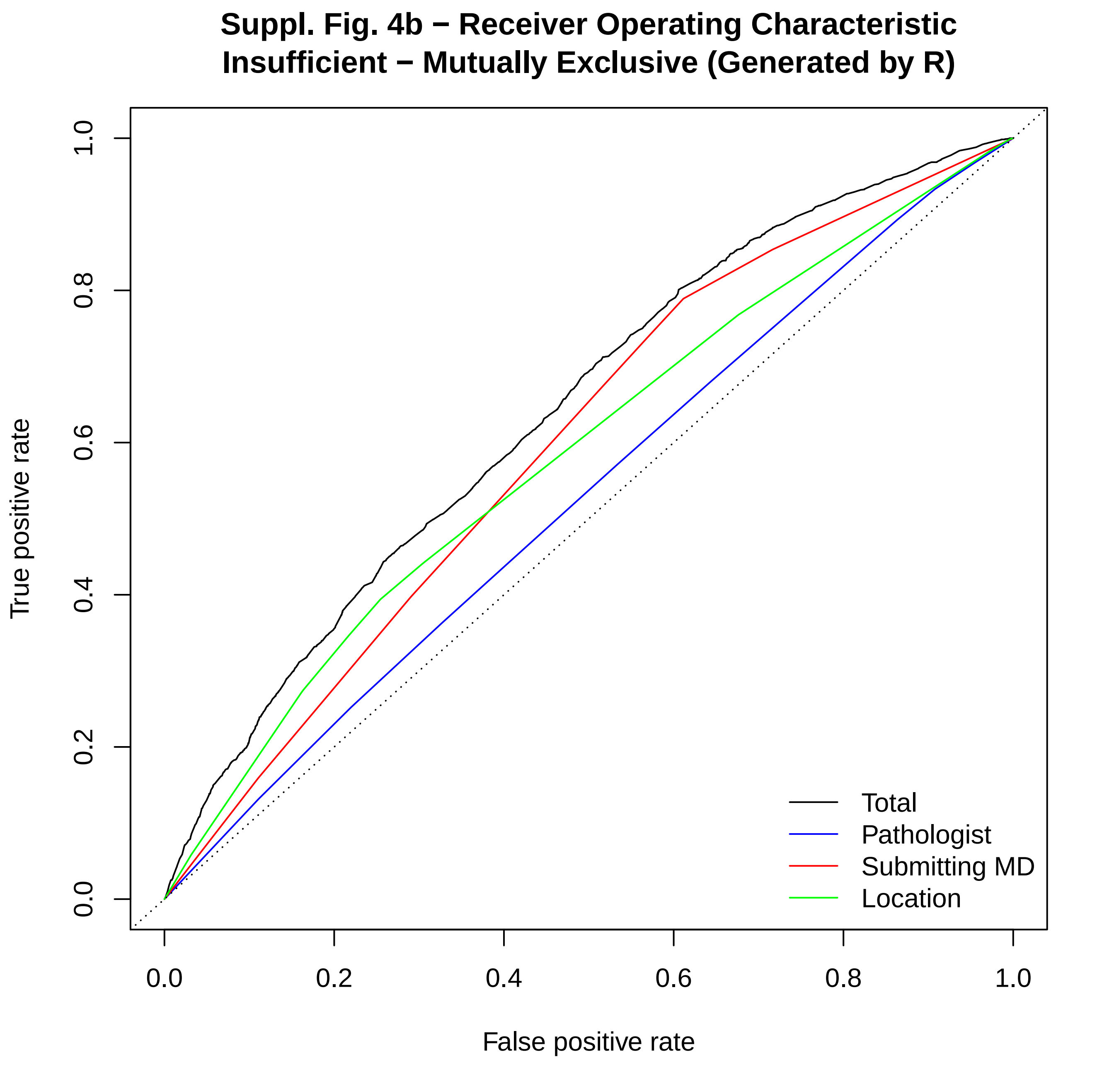
Suppl. Fig. 4b − Receiver Operating Characteristic
Insufficient − Mutually Exclusive (Generated by R)
True positive rate
False positive rate
0.0
0.2
0.4
0.6
0.8
1.0
Total
Pathologist
Submitting MD
Location

Suppl. Fig. 5a − Receiver Operating Characteristic
Adenocarcinoma (Generated by R)

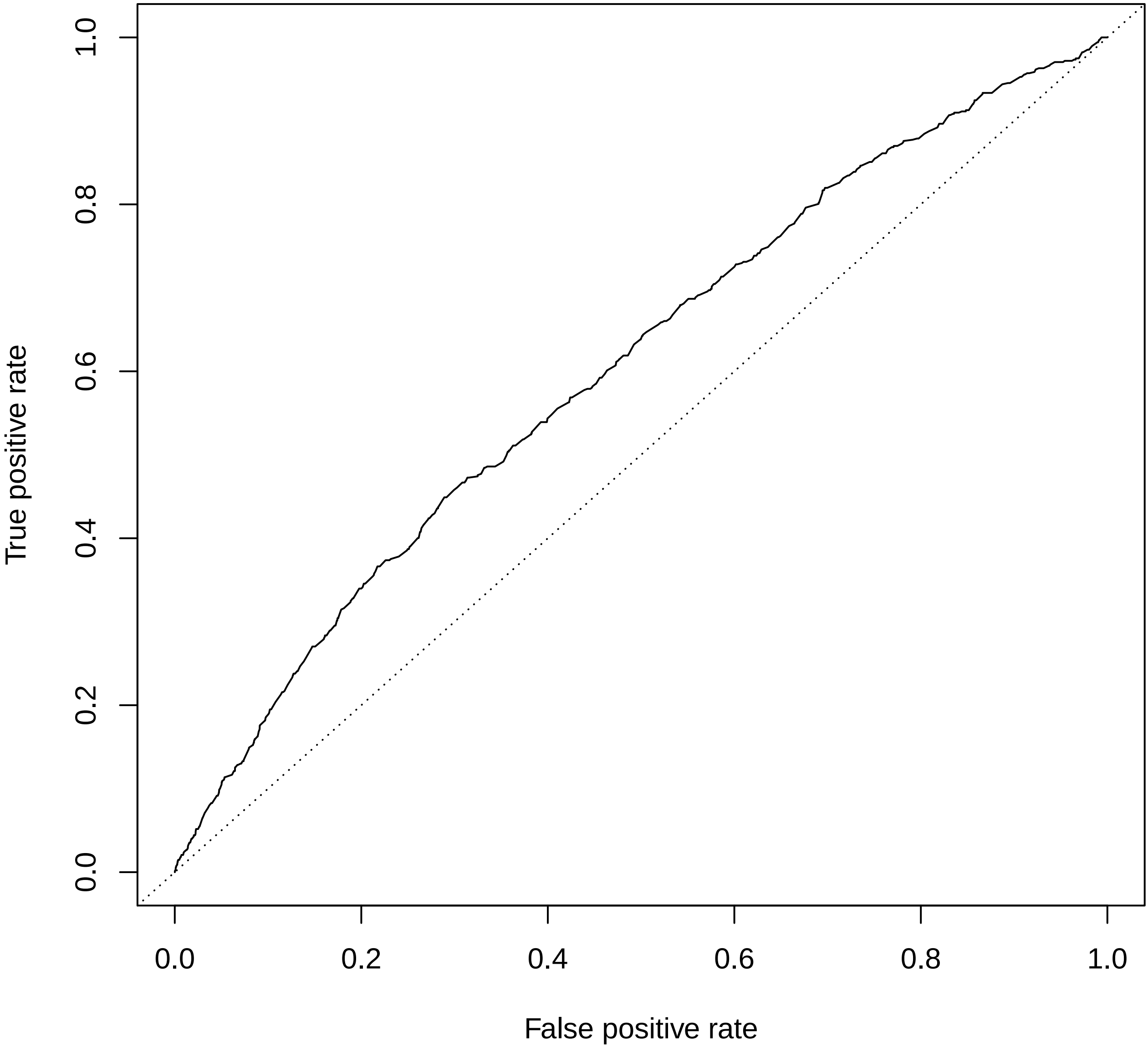

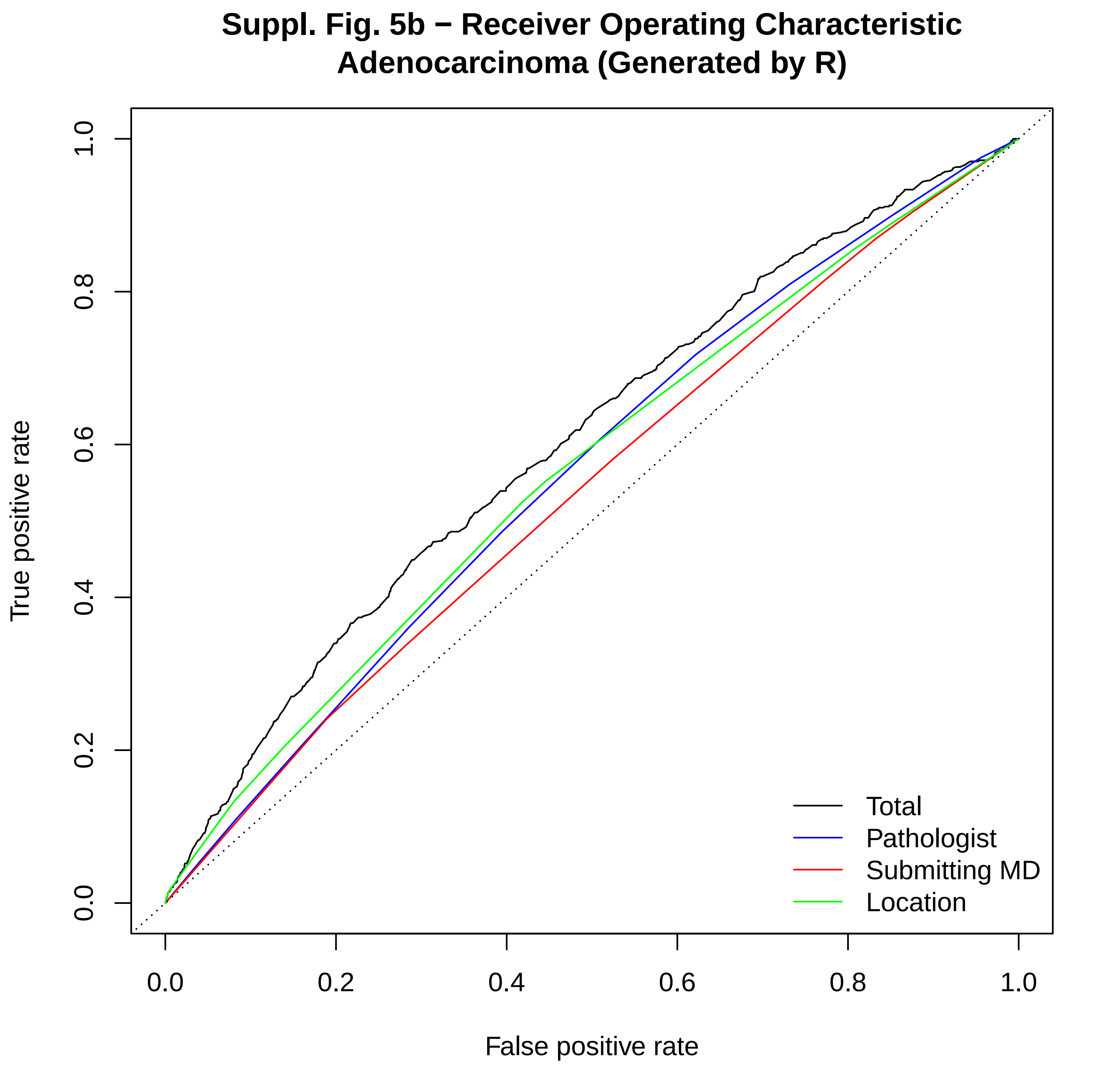
Suppl. Fig. 5b − Receiver Operating Characteristic
Adenocarcinoma (Generated by R)
True positive rate
False positive rate
0.0
0.2
0.4
0.6
0.8
1.0
Total
Pathologist
Submitting MD
Location

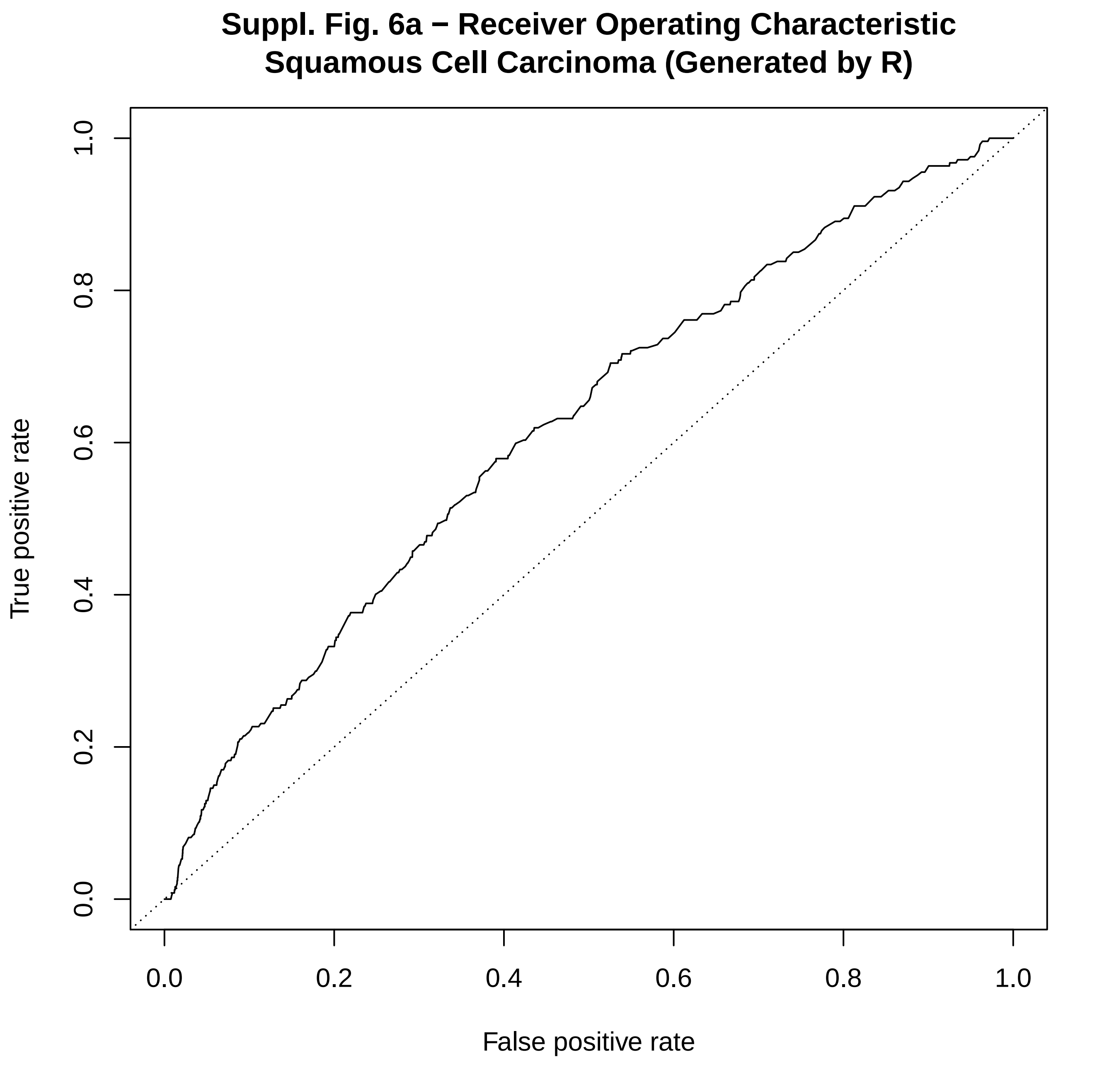
Suppl. Fig. 6a − Receiver Operating Characteristic
Squamous Cell Carcinoma (Generated by R)
True positive rate
0.0
0.2
0.4
0.6
0.8
1.0
False positive rate
0.0
0.2
0.4
0.6
0.8
1.0

Suppl. Fig. 6b − Receiver Operating Characteristic
Squamous Cell Carcinoma (Generated by R)

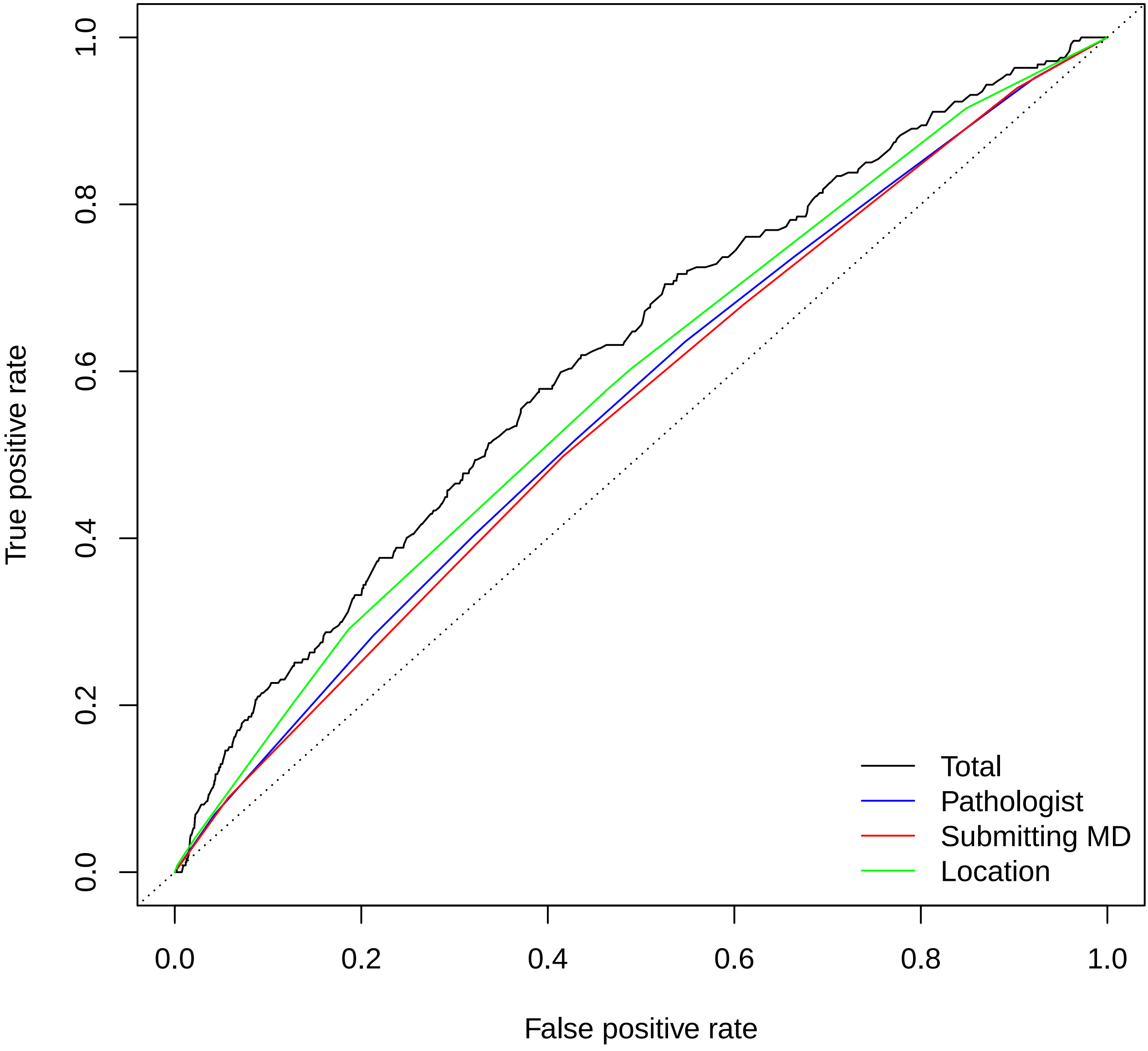

Suppl. Fig. 7a − Receiver Operating Characteristic
Small Cell Carcinoma (Generated by R)

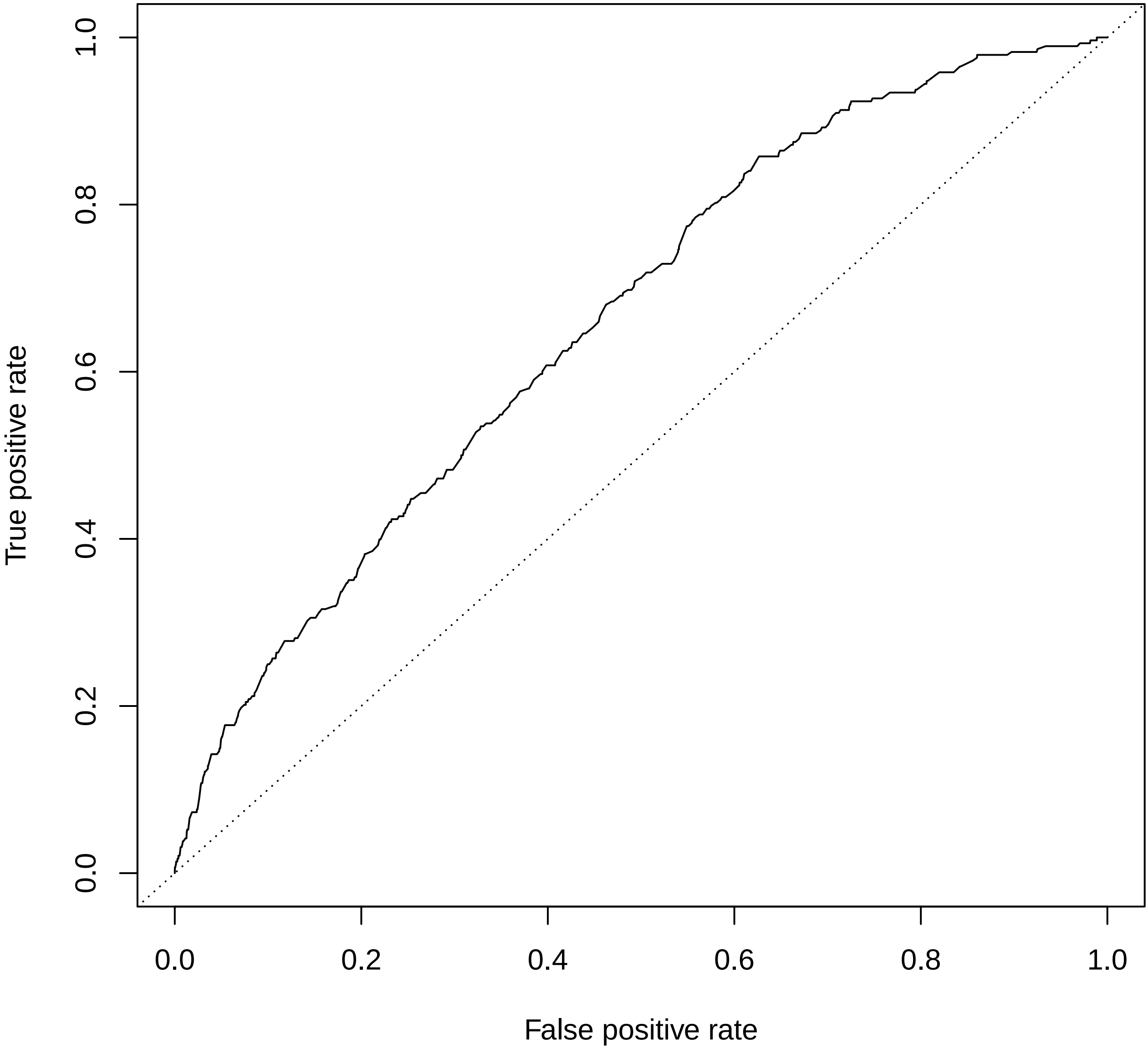

Suppl. Fig. 7b − Receiver Operating Characteristic Small Cell Carcinoma (Generated by R)

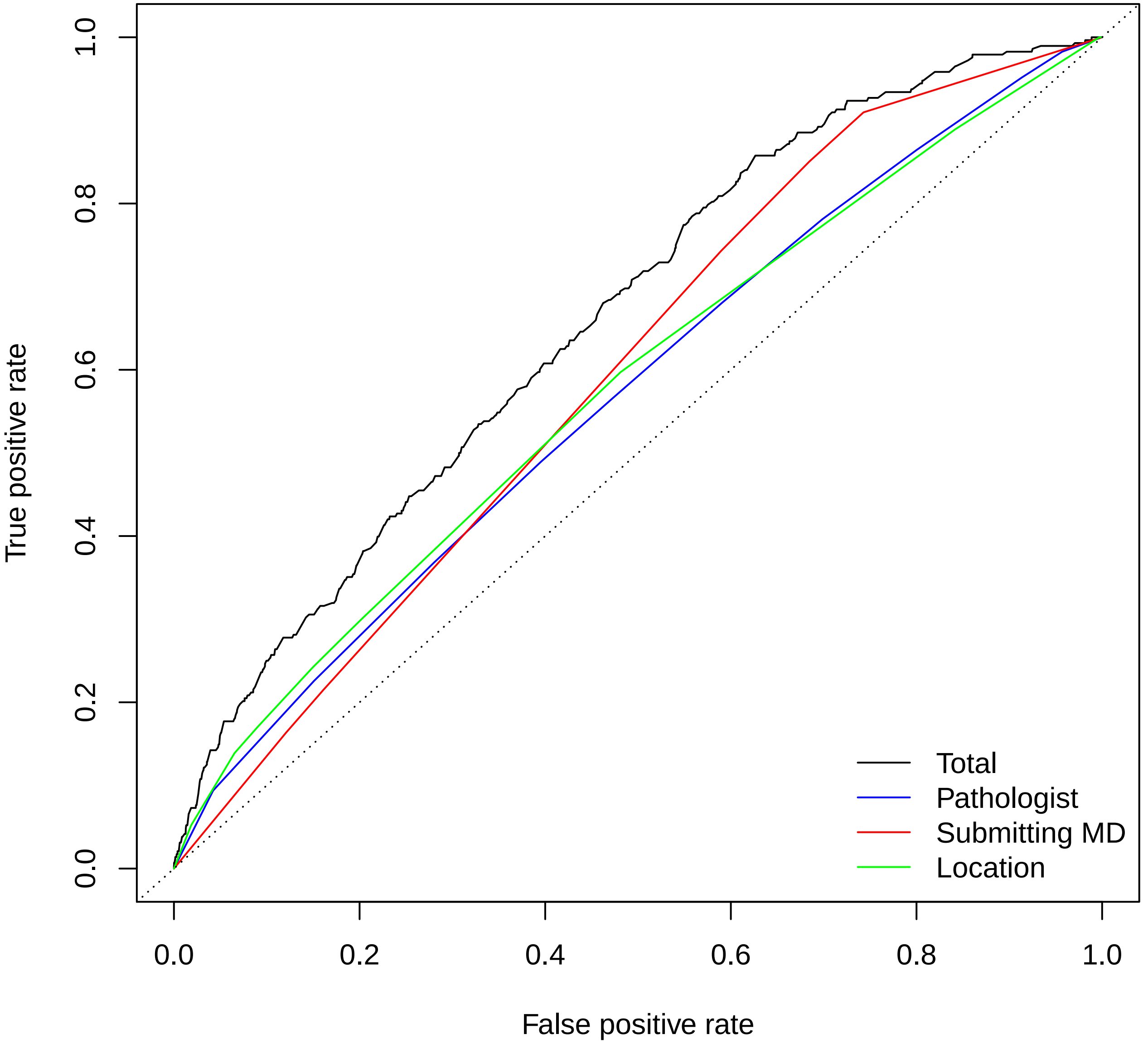

Suppl. Fig. 8a − Receiver Operating Characteristic Non−Small Cell Carcinoma−NOS (Generated by R)

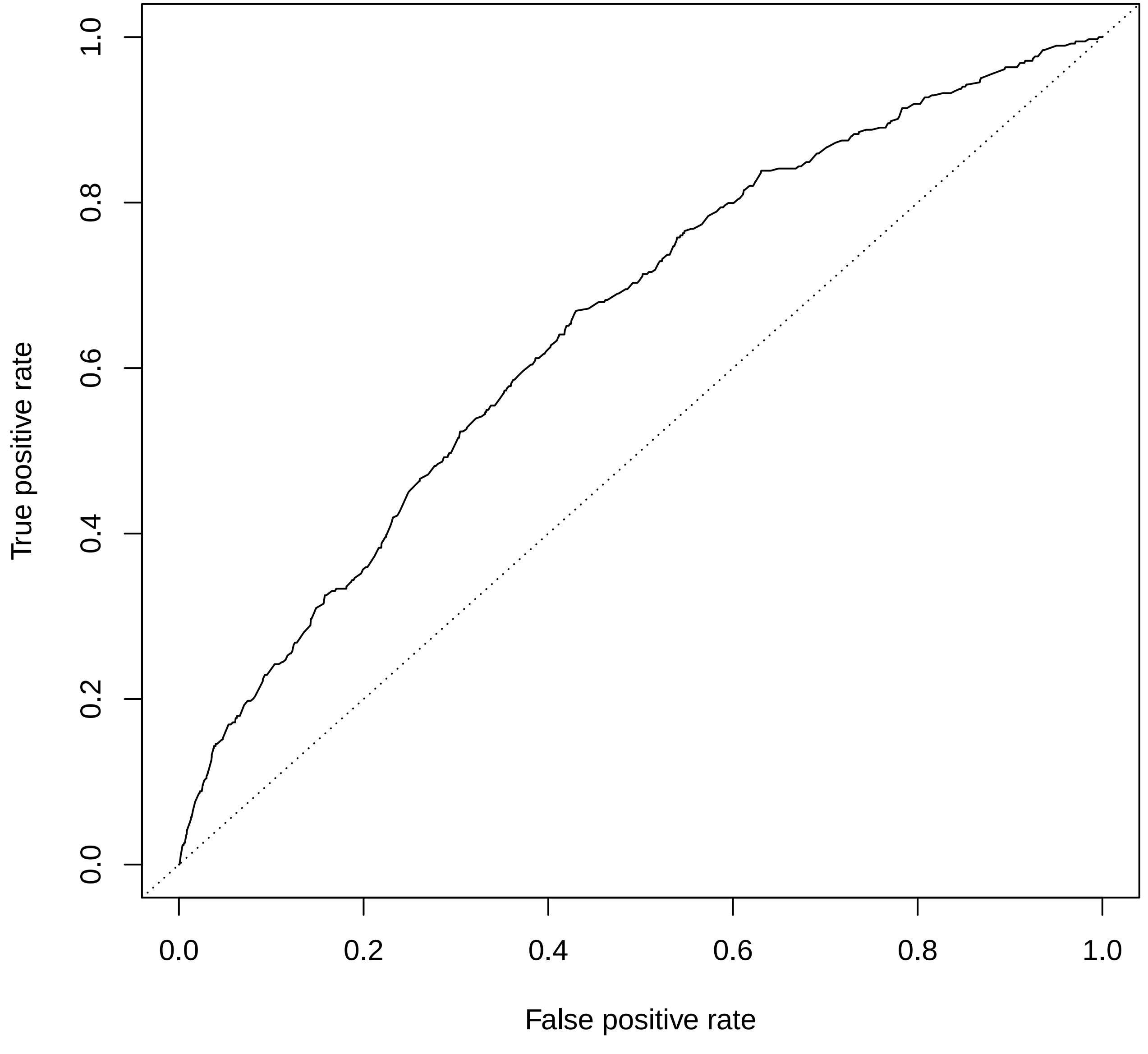

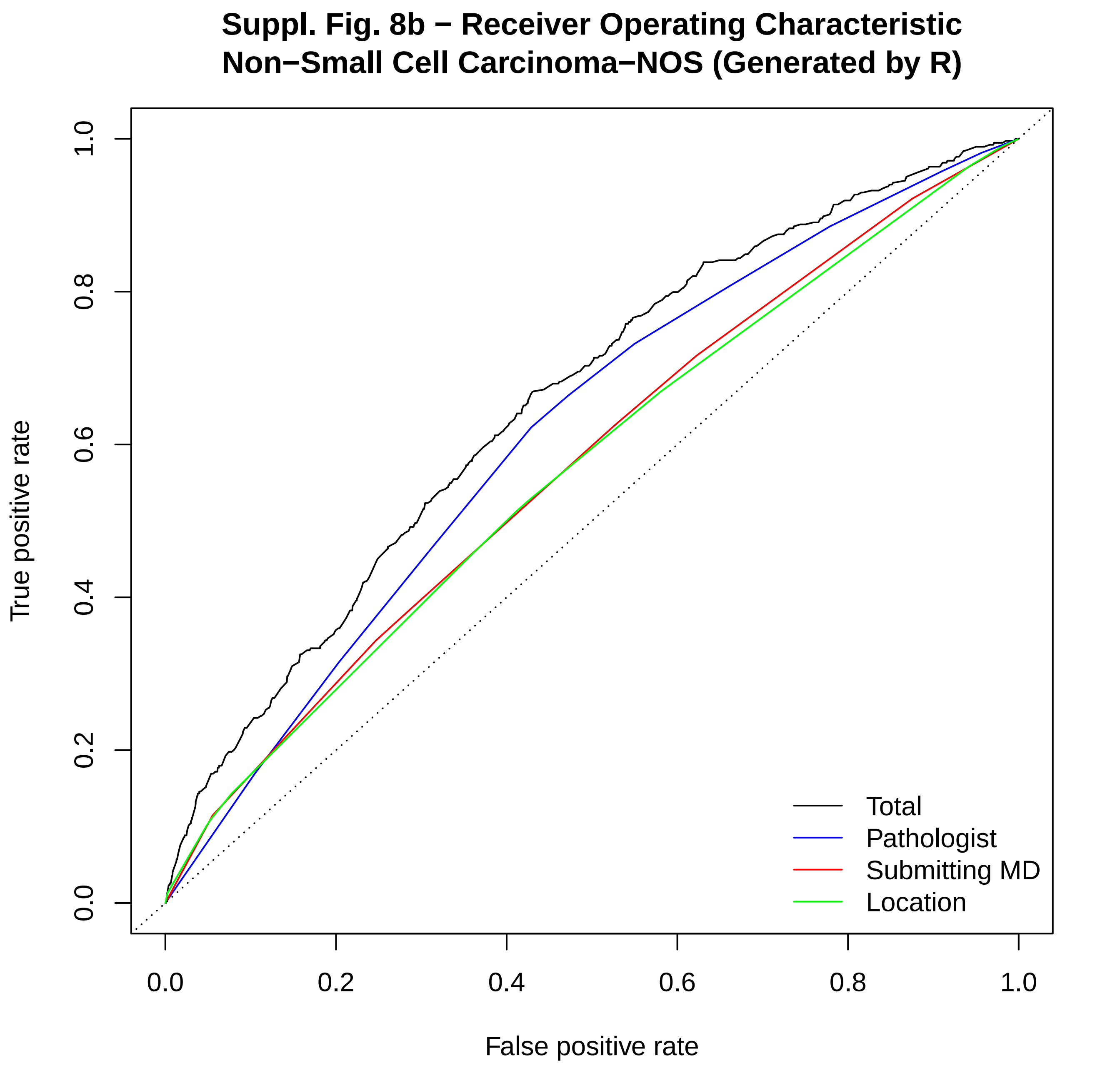
Suppl. Fig. 8b − Receiver Operating Characteristic
Non−Small Cell Carcinoma−NOS (Generated by R)
True positive rate
False positive rate
0.0
0.2
0.4
0.6
0.8
1.0
Total
Pathologist
Submitting MD
Location
