# Supplementary material for: The physician factor and anatomical site in 8846 consecutive mediastinal lymph node aspirations in a cross-sectional study
Source: Sci Rep. 2023 Jan 31;13:1784. doi: 10.1038/s41598-022-26962-w (PMC9889352; doi:10.1038/s41598-022-26962-w)
Supplement: Supplementary file 6 — Supplementary Information 6. [file 41598_2022_26962_MOESM6_ESM.docx]

**Table S1: Lymph node cohort by anatomical location.**

**Number of Specimens by Station**

| **Station** | **Number of Cases** | **Fraction of Cases** |
| --- | --- | --- |
| Station 7 | 3101 | 0.351 |
| Station 4R | 2453 | 0.277 |
| Station 4L | 1289 | 0.146 |
| Station 10R | 497 | 0.056 |
| Station 2R | 435 | 0.049 |
| Station 11R | 357 | 0.040 |
| Station 11L | 229 | 0.026 |
| Station 10L | 122 | 0.014 |
| Station 8R | 43 | 0.005 |
| Station 2L | 40 | 0.005 |
| Station 3P | 25 | 0.003 |
| Station 12R | 23 | 0.003 |
| Station 8 | 18 | 0.002 |
| Station 1R | 18 | 0.002 |
| Station 4 | 16 | 0.002 |
| Station 12L | 14 | 0.002 |
| Station 9 | 10 | 0.001 |
| Station 5 | 8 | 0.001 |
| Station 3 | 4 | 0.000 |
| Station 2P | 3 | 0.000 |
| Station GH | 2 | 0.000 |
| Station 6 | 1 | 0.000 |
| Station 8L | 1 | 0.000 |
| Station CEL | 1 | 0.000 |
| Missing | 138 * | 0.016 |
| *All cases* | *8846 *** | *1.000* |

* Two cases were labelled with two nodal stations each.

** The total number of cases is 8846 ‘*All cases*’. The total number of nodal stations is 8848. The difference (8846 versus 8848) comes from the two cases that were labelled with two nodal stations.

GH = gastrohepatic; CEL = celiac trunk
